# Gut microbiota metabolites impact immunologic responses to antiretroviral therapy in HIV-infected men who have sex with men

**DOI:** 10.1186/s40249-025-01291-y

**Published:** 2025-03-18

**Authors:** Anping Feng, Heping Zhao, Chunting Qiu, Dan Luo, Hao Wu, Xiaojun Meng, Linghua Li, Huachun Zou

**Affiliations:** 1 School of Public Health (Shenzhen), Sun Yat-Sen University, Shenzhen, China; 2 Infectious Disease Center, Guangzhou Eighth People’s Hospital, Guangzhou Medical University, No 8 Huaying Road, Guangzhou, 510060 Guangdong China; 3 Department of Infectious Diseases, Tianjin Second People’s Hospital, Tianjin, 300192 China; 4 Guangzhou Center for Disease Control and Prevention, Guangzhou, China; 5 The Affiliated Wuxi Center for Disease Control and Prevention of Nanjing Medical University, Wuxi, 214023 Jiangsu China; 6 School of Public Health, Fudan University, Room 435, Bld #8, 130 Dongan Road, Xuhui District, Shanghai, 200032 China; 7 School of Public Health, Southwest Medical University, Luzhou, China; 8 Kirby Institute, University of New South Wales, Sydney, Australia

**Keywords:** People living with HIV, Immunologic response, Metabolites, Men who have sex with men, China

## Abstract

**Background:**

The association between gut microbial metabolites and immunologic non-response among people living with HIV (PLHIV) receiving antiretroviral therapy (ART) has not been well established. We aimed to characterize gut microbial metabolites among HIV-infected men who have sex with men (MSM) with different immunologic responses.

**Methods:**

We recruited HIV-infected MSM from Guangzhou Eighth People’s Hospital and HIV-uninfected MSM (healthy controls, HC) from a local MSM community-based organization in Guangzhou between June and October 2021. HIV-infected MSM were grouped into good immunological responders (GIR) (CD4 + T cell count ≥ 350 cells/μl) and poor immunological responders (PIR) (CD4 + T cell count < 350 cells/μl) after 24 months of ART treatment. Online questionnaires and stool samples were collected. Microbial metabolites in stool were obtained through ultra-performance liquid chromatography coupled to a tandem mass spectrometry (UPLC-MS/MS) system. Differential metabolites were identified and analyzed using the Kruskal–Wallis test, followed by pairwise comparisons with the Wilcoxon rank-sum test. The least absolute selection and shrinkage operator was used to select potential metabolites biomarkers.

**Results:**

A total of 51 HC, 56 GIR, and 42 PIR were included. No statistically significant differences were observed in the median time since HIV diagnosis and ART duration between GIR and PIR. Among the 174 quantified metabolites, 81 significantly differed among HC, GIR, and PIR (*P* < 0.05). Among differential metabolites, indole-3-propionic acid significantly decreased from HC (11.39 nmol/g) and GIR (8.16 nmol/g) to PIR (6.50 nmol/g). The pathway analysis showed that tryptophan metabolism differed significantly between GIR and PIR (*P* < 0.05). Four potential metabolites biomarkers (dimethylglycine, cinnamic acid, 3-hydroxyisovaleric acid, and propionic acid) that distinguish GIR and PIR were identified, and the corresponding area under the curve based on potential biomarkers was 0.773 (95% *CI*: 0.675–0.871).

**Conclusions:**

This study identified significant differences in gut microbial metabolites among HIV-infected MSM with different immunologic responses. These results indicate the potential of gut microbial metabolites as novel disease progression markers and therapeutic targets.

**Graphical Abstract:**

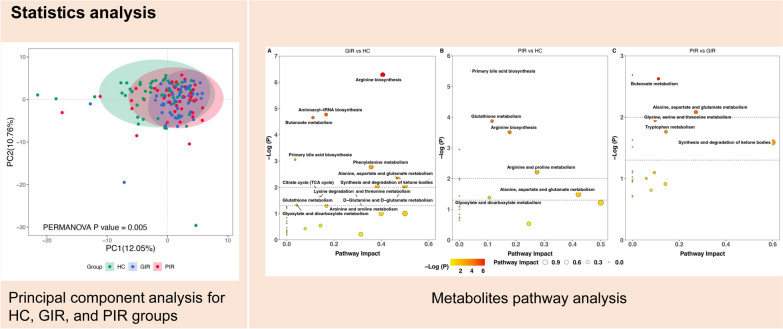

**Supplementary Information:**

The online version contains supplementary material available at 10.1186/s40249-025-01291-y.

## Background

With the wide use of antiretroviral therapy (ART), AIDS has become a manageable chronic disease [1]. The CD4 + T cell counts and HIV viral load are routinely used to monitor ART responses and HIV disease progression. However, about 10–40% of people living with HIV (PLHIV) fail to restore their CD4 + T cell count to a satisfactory level despite full virologic suppression for several years, a condition known as immunologic non-response [2]. Existing studies found that the mortality rate among immunologic non-responders was significantly higher than that of immunologic responders [3, 4].

Increasing evidence shows that the gut microbiota profile in PLHIV differs from that of HIV-uninfected individuals [5, 6], and the changes in gut microbiota may affect the recovery of immune function in PLHIV [7, 8]. It is widely recognized that the significant biological effects of gut microbiota are primarily mediated by microbial metabolites [9]. Previous studies have demonstrated that short-chain fatty acids play a crucial role in regulating the function of immune cells [10, 11]. Additionally, recent studies have identified several altered metabolites in PLHIV undergoing ART [12, 13]. The underlying mechanisms of immunologic non-response among PLHIV are likely multifactorial, including aberrant immune activation, inadequate thymic output, reduced hematopoiesis of bone marrow, and HIV reservoirs [2, 14–16]. Metabolomics, which involves the large-scale analysis of small molecule metabolites in biological specimens, has emerged as a promising diagnostic tool in detection of metabolic biomarkers for various diseases, including cancer [17], cardiovascular disease [18], and diabetes [19]. Applying metabolomics to investigate potential biomarkers associated with immunologic non-response in PLHIV is valuable for identifying novel disease progression markers and therapeutic targets.

Men who have sex with men (MSM) are disproportionately affected by HIV worldwide [20]. A systematic review of 38 low- and middle-income countries showed that MSM have a 19.3 times higher risk of HIV infection than the general population [21]. In many countries, new HIV infections are predominately found among MSM [22]. While some studies have investigated the role of gut microbial metabolites in immune recovery among PLHIV initiating ART [23–25], their findings have been inconsistent, and these studies did not strictly control sex and sexual orientation. Previous research has indicated that MSM exhibit a distinct fecal microbiome compared to non-MSM, independent of HIV infection status [26, 27]. These reports raise questions about whether earlier observations of HIV-related alterations in the gut microbiota may have captured microbial variations related to MSM or a combined effect of both. Given the significant burden of HIV among MSM, our study aimed to investigate variations in gut microbial metabolites among HIV-infected MSM with different immunologic responses after ART.

## Methods

### Study design and participants

HIV-infected MSM with an HIV viral load < 20 copies/ml after more than 24 months of ART were recruited from the Guangzhou Eighth People’s Hospital between June and October 2021. All field questionnaire surveys and biological samples collections (including blood and stool samples) were conducted in the morning. Participants were stratified into good immunological responders (GIR) and poor immunological responders (PIR) depending on the recovery of CD4 + T cell count (≥ 350 and < 350 cells/μl respectively) [28, 29]. The inclusion criteria were (1) biologically male, (2) 18–50 years of age, (3) having had sex with men, (4) CD4 + T cell count < 350 cells/μl at ART initiation, and (5) having received ART for at least 24 months. HIV-uninfected MSM (Healthy Controls, HC) were enrolled through a local MSM community-based organization in Guangzhou.

The China Health and Nutrition Survey indicates that the dietary patterns in China are currently transitioning from traditional to Western dietary patterns [30]. Different dietary patterns, such as vegetarian diet and ketogenic diet, can profoundly alter the composition and function of the gut microbiome [31]. Participants were excluded if they: (1) had a history of using antibiotics, probiotics, prebiotics, or symbiotics in the past six months; (2) had undergone gastrointestinal surgery; (3) adhered to special diet habits, including vegetarianism, low-carbon diet, and ketogenic diet in the past six months; (4) had active inflammatory diseases that may influence gut microbiota, such as inflammatory bowel disease, hypertension, diabetes, malignant tumor, and others.

For the HC group, an initial screening was conducted via an online questionnaire covering various topics such as demographic characteristics, sexual orientation, health history, dietary habits, and bowel habits. Additionally, participants were required to take a rapid, on-site HIV test [SD Bioline HIV/Syphilis Duo test (Standard Diagnostics)] using a finger stick blood sample. Only those with a negative HIV test result were included in the study. For the GIR and PIR groups, data were obtained from medical records, including self-reported HIV acquisition route, CD4 + T cell count, CD8 + T cell count, HIV viral load, biochemical indices, the date of HIV diagnosis, and ART initiation date. Furthermore, demographic characteristics, sexual orientation, dietary habits, bowel habits, and other relevant lifestyle factors were collected through a field questionnaire. Missing values were imputed using the median for continuous variables and the mode for categorical variables.

This study received ethics approval from the Ethics Review Board of the School of Public Health (Shenzhen), Sun Yat-sen University (SYSU-SPH2020047). Signed informed consent was obtained from each participant at enrollment. To protect participants’ privacy, identifying information including name, phone number, address, and ID number were not collected.

### Measures

Targeted metabolomic analysis of stool samples was performed by Metabo-Profile Biotechnology (Shanghai, China) according to the methodology described in previous studies [32]. Fresh stool samples were collected in sterile tubes from each participant. Within 4 h, the stool samples were transferred to the laboratory in an ice bath, and stored at −80 ℃. To identify microbial metabolites that potential to play active roles in immunologic response, we targeted a panel of metabolites that participate in multiple metabolic pathways related to host-gut microbiota co-metabolism [33]. Each stool sample (approximately 5 mg) was homogenized with 25 μl of water and zirconium oxide beads for 3 min. Then, 120 μl of methanol containing an internal standard was added. The sample was centrifuged at 18,000×*g* for 20 min after homogenizing for another 3 min. The supernatant (20 μl) was transferred to a 96-well plate, and following procedures were performed on the Eppendorf epMotion Workstation (Eppendorf Inc., Humburg, Germany). In each well, 20 μl of freshly prepared derivative reagents were added, and the plate was sealed and derivatized (30 °C for 60 min). After derivatization, 330 μl of ice-cold 50% methanol solution was added to dilute the sample. The plate was then stored at −20 °C for 20 min, followed by 4000×*g* centrifugation at 4 °C for 30 min. Finally, 135 μl of the supernatant was transferred to a new 96-well plate, with 10 μl of internal standard added to each well. Aliquots of metabolite extracts were mixed to prepare the pooled quality control (QC) sample, which was prepared along with the study samples and run after every 10 study samples.

Microbial metabolites were quantitated in ultra-performance liquid chromatography coupled with tandem mass spectrometry (UPLC_MS/MS) system (ACQUITY UPLC-Xevo TQ-S; Waters Corp., Milford, MA, USA). We used HPLC columns, including an ACQUITY HPLC BEH C18 1.7 μm VanGuard precolumn (2.1 mm × 5 mm) and an ACQUITY HPLC BEH C18 1.7 μm analytical column (2.1 mm × 100 mm), for chromatographic separation of stool samples. The column temperature was set at 40 °C, and the sample manager temperature was maintained at 10 °C. The mobile phases consisted of solvent A (water with 0.1% formic acid) and solvent B (acetonitrile/IPA, 70:30). The gradient conditions were as follows: 0–1 min (5% B), 1–11 min (5–78% B), 11–13.5 min (78–95% B), 13.5–14 min (95–100% B), 14–16 min (100% B), 16–16.1 min (100–5% B), and 16.1–18 min (5% B). The flow rate was 0.40 ml/minute, and the injection volume was 5.0 µl. The mass spectrometer settings were as follows: capillary: 1.5 kV (ESI+), 2.0 kV (ESI–), source temperature: 150 °C, desolvation temperature: 550 °C, and desolvation gas flow: 1000 L/h. Details of MRM transitions for 3-NPH derivatized metabolites, along with their linearity range, precision, and accuracy, are listed in Table S1. The raw data generated by UPLC_MS/MS were processed by TMBQ software (v1.0, Metabo-Profile, Shanghai, China) for peak integration, calibration, and quantitation as described elsewhere [32].

### Statistical analysis

The sociodemographic and clinical characteristics of participants were presented as frequency (%) for categorical variables and median [interquartile range (IQR)] for continuous variables. Principal component analysis (PCA) was performed to identify structure in the distribution of microbial metabolites across different groups. The *P* value in PCA was calculated using permutational multivariate analysis of variance (PERMANOVA) with distance matrices. Differential metabolites among the three groups were assessed by the Kruskal–Wallis test. Pairwise comparisons of differential metabolites between the groups were evaluated using the Wilcoxon rank-sum test and presented in volcano plots. All *P*-values were FDR-corrected using the BH method. Metabolomics data often exhibit high dimensionality and multicollinearity. The least absolute selection and shrinkage operator (LASSO) is a regularization technique for managing overfitting and performing variable selection. It adds the L1 norm of the coefficients as a penalty term to the loss function, thereby constraining the coefficients [34]. LASSO has been applied in various metabolomics data analyses to perform necessary data reduction prior to building the model [35, 36]. A previous study, using multiple machine learning methods to identify disease-related metabolic biomarkers, demonstrated that the LASSO regression model exhibited the best predictive performance, while linear regression and ridge regression models performed poorly [37]. All significantly altered metabolites were screened using LASSO with tenfold cross-validation to define the λ parameter that resulted in the minimum mean squared error of the regression model, employing the “glmnet” package in R [38]. Metabolites with a non-zero β-coefficient in at least one of the ten LASSO models were included for further analysis. Logistic models were built for metabolite selection in the LASSO method, and receiver operating characteristic (ROC) curves were conducted to illustrate the performances of models. Statistical differences were determined at *P* < 0.05. Sensitivity analysis was conducted to investigate whether rectal douching is associated with altered gut microbiota metabolites. All statistical analyses were performed using R version 4.2.1 (R Core Team, Vienna, Austria). To explore the functions of significantly altered metabolites for each paired group, pathway analysis was performed using the Kyoto Encyclopedia of Genes and Genomes (KEGG) Database in MetaboAnalyst 5.0 (University of Alberta, Edmonton, Canada).

## Results

### Participants’ characteristics

A total of 149 participants were included in our study, including 51 HC, 56 GIR, and 42 PIR. Most of them were single (88.59%, 132/149) and homosexual (85.23%, 127/149). Additionally, 30.20% (45/149) reported rectal douching in the past 3 months. Participants in the HC group were younger than those in the GIR and PIR groups. PIR were more likely to report having no male sexual partners and using condoms in the past 6 months. No significant differences in BMI (*P* = 0.386), sexual orientation (*P* = 0.478), rectal douching (*P* = 0.223), defecation frequency (*P* = 0.055), and stool form (*P* = 0.673) were found among HC, GIR, and PIR. Time since HIV diagnosis and ART duration were similar between PIR and GIR. PIR reported lower CD4 + T cell count and CD8 + T cell count, as well as more advanced WHO stage (Table 1). No significant differences were observed between PIR and GIR regarding hepatitis B virus (HBV) co-infection rates (*P* = 0.114), and no individuals reported co-infections with hepatitis C virus (HCV) or cytomegalovirus (CMV). Among the HIV-infected MSM who reported their baseline ART regimens (97.95%, 96/98), all regimens included nucleoside reverse transcriptase inhibitors (NRTIs), and the majority of participants (86.46%, 83/96) received a combination of NRTIs and non-nucleoside reverse transcriptase inhibitors (NNRTIs) (Table S2).

**Table 1:** Demographics and clinical characteristics of HIV-infected and HIV-uninfected MSM

	Overall	HC	GIR	PIR	*P* value
*N*	149	51	56	42	
Age, Mean (*SD*)	32.07 (7.37)	27.66 (4.84)	32.45 (6.63)	36.93 (7.74)	< 0.001^*^
BMI, Mean (*SD*)	21.67 (2.85)	21.93 (2.99)	21.83 (3.11)	21.16 (2.26)	0.386
Marriage, *N* (%)					0.001^*^
Single	132 (88.59)	51 (100.00)	50 (89.29)	31 (73.81)	
Married	7 (4.70)	0 (0.00)	1 (1.79)	6 (14.29)	
Divorce	10 (6.71)	0 (0.00)	5 (8.93)	5 (11.90)	
Sexual orientation, *N* (%)					0.478
Homosexual	127 (85.23)	47 (92.16)	45 (80.36)	35 (83.33)	
Bisexual	14 (9.40)	2 (3.92)	7 (12.50)	5 (11.90)	
Uncertain	8 (5.37)	2 (3.92)	4 (7.14)	2 (4.76)	
Number of male sexual partners in the previous 6 months, *N* (%)					< 0.001^*^
0	60 (40.27)	5 (9.80)	28 (50.00)	27 (64.29)	
1	47 (31.54)	18 (35.29)	18 (32.14)	11 (26.19)	
≥ 2	42 (28.19)	28 (54.90)	10 (17.86)	4 (9.52)	
Sex role in the previous 6 months, *N* (%)					< 0.001^*^
Receptive only	32 (21.48)	14 (27.45)	14 (25.00)	4 (9.52)	
Insertive only	27 (18.12)	15 (29.41)	8 (14.29)	4 (9.52)	
Insertive and receptive	30 (20.13)	17 (33.33)	6 (10.71)	7 (16.67)	
Not applicable^a^	60 (40.27)	5 (9.80)	28 (50.00)	27 (64.29)	
Frequency of condom use in the previous 6 months, *N* (%)					< 0.001^*^
Sometimes	29 (19.46)	20 (39.22)	7 (12.50)	2 (4.76)	
Always	60 (40.27)	26 (50.98)	21 (37.50)	13 (30.95)	
Not applicable^a^	60 (40.27)	5 (9.80)	28 (50.00)	27 (64.29)	
Rectal douching in the previous 3 months, *N* (%)					0.223
Yes	45 (30.20)	20 (39.22)	14 (25.00)	11 (26.19)	
No	104 (69.80)	31 (60.78)	42 (75.00)	31 (73.81)	
Defecation frequency in the past month, *N* (%)					0.055
2–3 times per day	49 (32.89)	13 (25.49)	22 (39.29)	14 (33.33)	
1 time per day	91 (61.07)	31 (60.78)	33 (58.93)	27 (64.29)	
2–3 times per day	9 (6.04)	7 (13.73)	1 (1.79)	1 (2.38)	
Stool form in the past month, *N* (%)					0.673
Separate hard lumps	2 (1.34)	1 (1.96)	1 (1.79)	0 (0.00)	
Lumpy and sausage like	8 (5.37)	3 (5.88)	3 (5.36)	2 (4.76)	
A sausage shape with cracks in the surface	33 (22.15)	12 (23.53)	12 (21.43)	9 (21.43)	
Like a smooth, soft sausage or snake	62 (41.61)	15 (29.41)	25 (44.64)	22 (52.38)	
Soft blobs with clear-cut edges	35 (23.49)	17 (33.33)	11 (19.64)	7 (16.67)	
Mushy consistency with ragged edges	9 (6.04)	3 (5.88)	4 (7.14)	2 (4.76)	
Baseline WHO stage, *N* (%)					0.004^*^
II	2 (2.04)		2 (3.57)	0 (0.00)	
III	89 (90.82)		54 (96.43)	35 (83.33)	
IV	7 (7.14)		0 (0.00)	7 (16.67)	
Time since HIV diagnosis, year, Median (IQR)	4.25 (3.00, 6.15)		4.20 (2.95, 6.70)	4.45 (3.02, 5.70)	0.530
ART duration, y, Median (IQR)	3.90 (2.55, 5.40)		4.05 (2.85, 6.00)	3.65 (2.42, 4.88)	0.219
Baseline CD4 + T cell count (cells/μl), Median (IQR)	153.50 (37.50, 274.00)		248.00 (163.75, 306.00)	36.00 (18.25, 100.25)	< 0.001^*^
Baseline CD8 + T cell count (cells/μl), Median (IQR)	695.00 (470.25, 1096.00)		924.00 (566.50, 1206.00)	596.00 (390.50, 751.75)	< 0.001^*^
Latest CD4 + T cell count (cells/μl), Median (IQR)	392.50 (211.75, 536.75)		527.50 (440.75, 595.25)	191.50 (132.75, 235.25)	< 0.001^*^
Latest CD8 + T cell count (cells/μl), Median (IQR)	707.00 (551.00, 926.25)		736.50 (583.75, 941.00)	675.50 (416.50, 904.00)	0.160

*HC* healthy controls, *GIR* good immunological responders, *PIR* poor immunological responders, *BMI* body mass index, *IQR* interquartile range, *WBC* white blood cell, *PLT* platelet, *Hb* hemoglobin, Scr serum creatinine, *TG* triglyceride, *TC* total cholesterol, *Glu* glucose, *AST* aspartate aminotransferase, *ALT* alanine aminotransferase, *TBIL* total bilirubin, *SD* standard deviation

^a^ If, over the past 6 months, the participants did not have any male sexual partners, the specific sexual roles and frequency of condom use during that time were categorized as unknown

^*^Showed significant differences between different groups

### Metabolite profiling

**Fig. 1:**
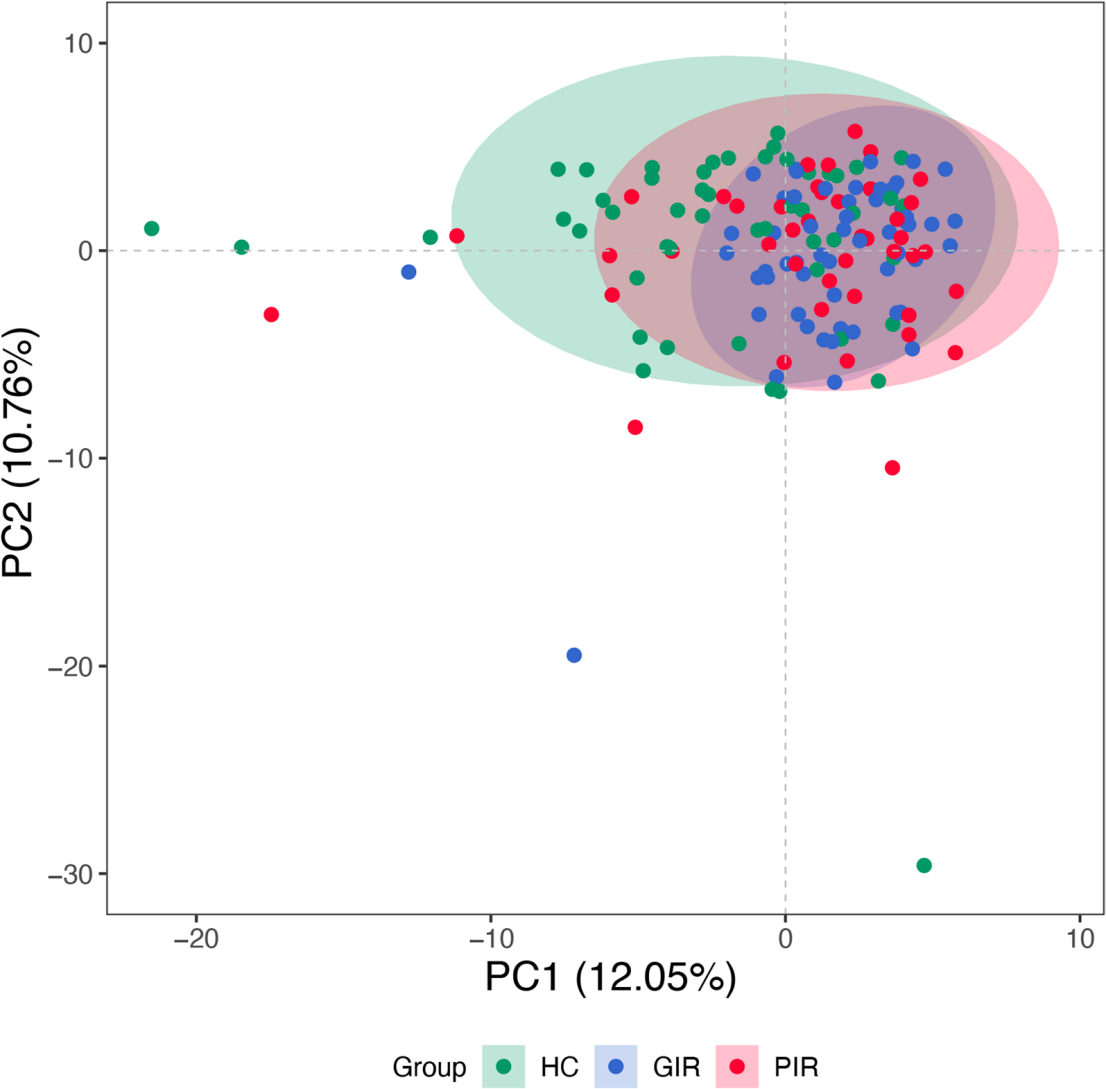
Principal component analysis for HC, GIR, and PIR groups. HC, healthy controls; PIR, poor immunological responders; GIR, good immunological responders; PC, principal components

A total of 174 metabolites were quantified from stool samples of the 149 included participants. Unsupervised scatter plots generated by PCA scores revealed discernible distinctions in metabolite profiles among HC, GIR, and PIR, subsequently confirmed by PERMANOVA (*P* = 0.005) (Fig. 1). No significant differences were found between MSM who experienced rectal douching in the previous 3 months and those who did not (Figure S1).

### Altered metabolites and metabolic pathways

**Fig. 2:**
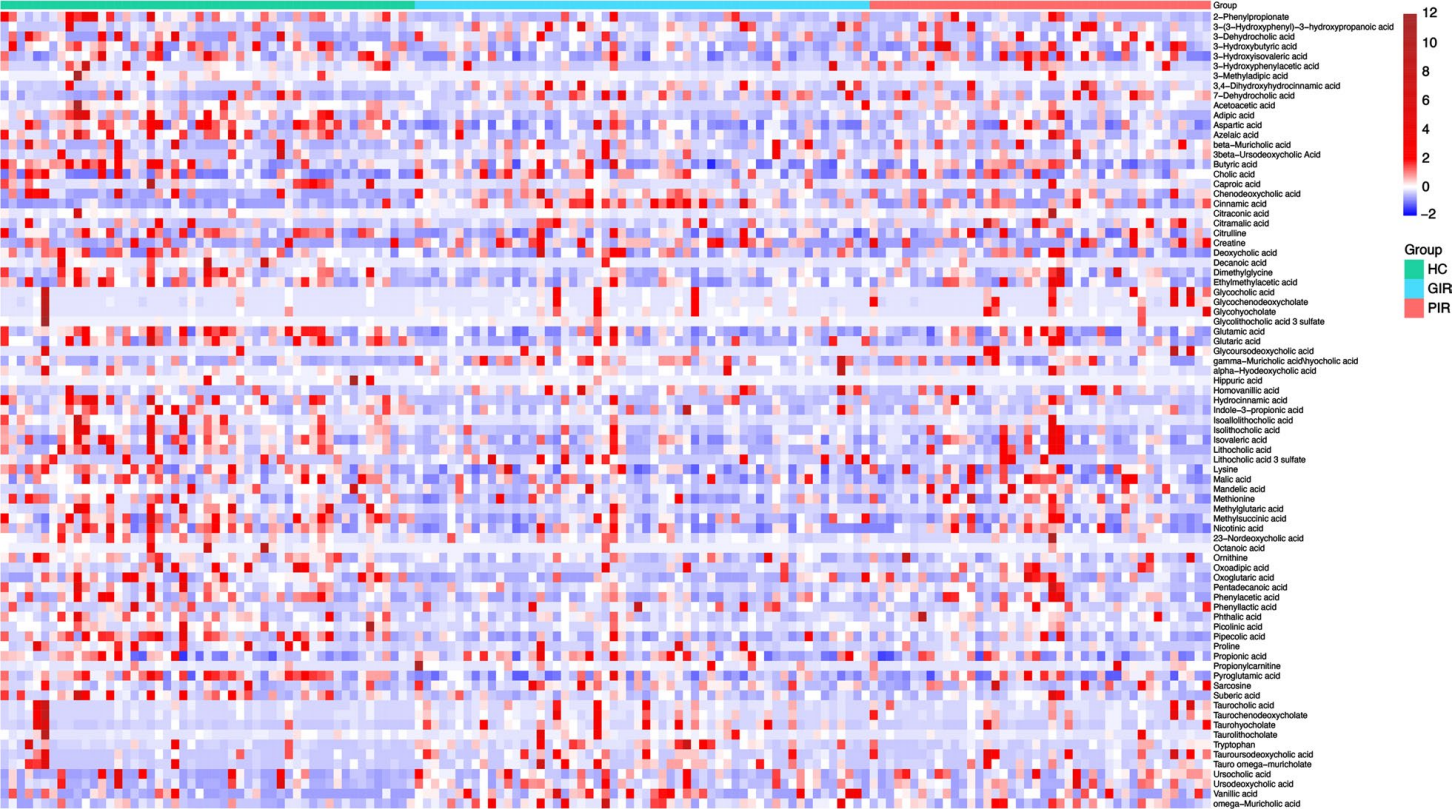
*Z*-score heatmap of 81 significantly altered metabolites among HC, GIR, and PIR. Significance of altered metabolites were determined using the Kruskal–Wallis test with a cut-off of *P* < 0.05. HC, healthy controls; PIR, poor immunological responders; GIR, good immunological responders

**Fig. 3:**
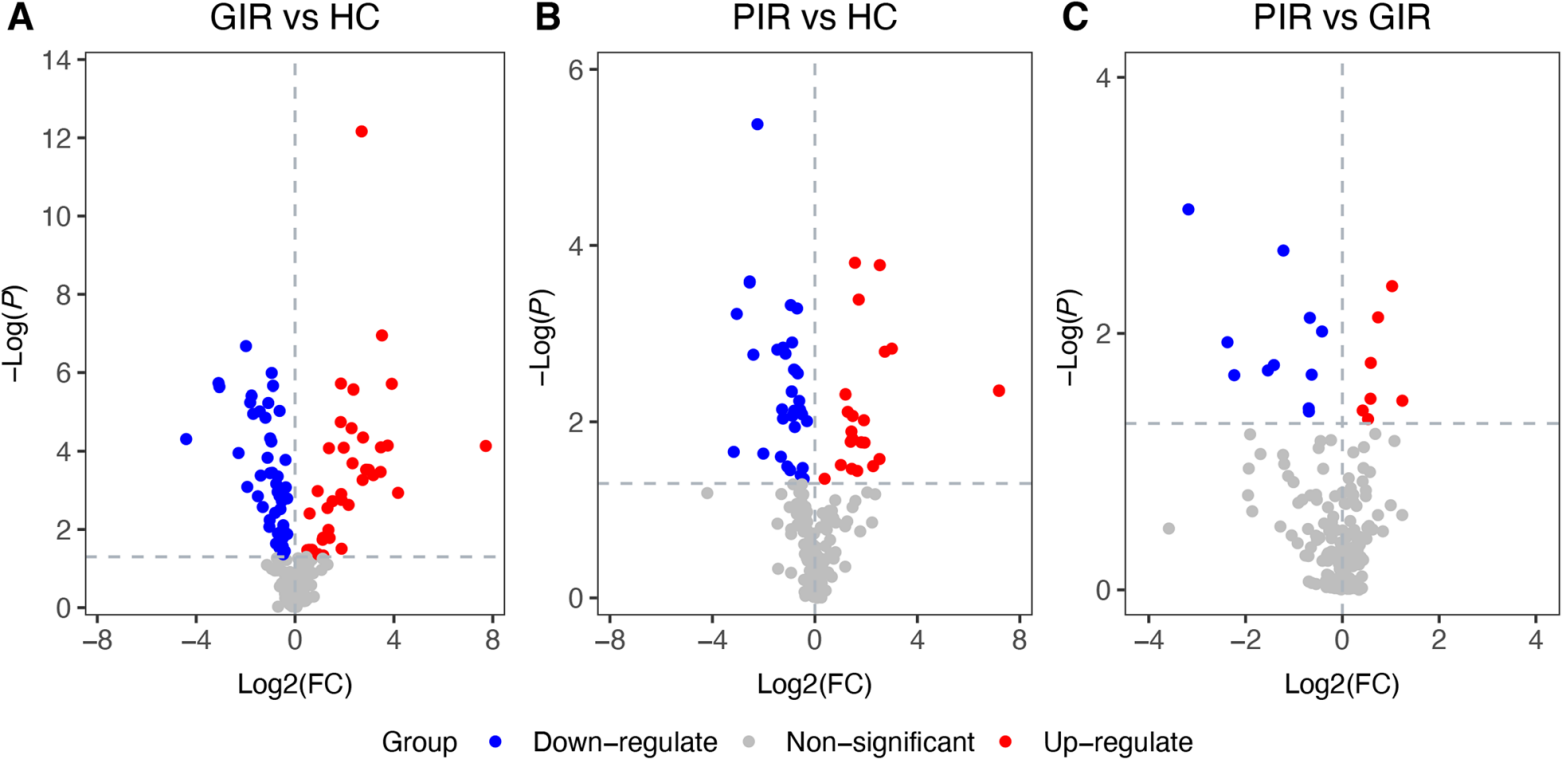
Volcano plots of significantly altered metabolites between groups. The significant metabolites highlighted in the volcano plot were defined as having a *P* value < 0.05 (Wilcoxon rank-sum test) and a fold change ≠ 1. Fold Change (FC) measures the ratio of metabolite concentrations between two groups. log2FC represents the magnitude and direction of concentration change (upregulation or downregulation). The *P* value tests whether the difference in metabolite concentration between two groups is statistically significant. **A** Compared to HC, a 88 metabolites showed significant differences, with 39 up-regulated and 49 down-regulated in the GIR group. **B** Compared to HC, 53 metabolites showed significant differences, with 21 up-regulated and 32 down-regulated in the PIR group. **C** Compared to GIR, 18 metabolites showed significant differences, with 7 up-regulated and 11 down-regulated in the PIR group. HC, healthy controls; PIR, poor immunological responders; GIR, good immunological responders

A total of 81 differential metabolites were identified among HC, GIR, and PIR (Fig. 2; Table S3). Notably, indole-3-propionic acid significantly decreased from HC and GIR to PIR, suggesting a potential role in immune regulation. A totlal of 88 metabolites were differentially abundant (*P* < 0.05) (Fig. 3A; Table S4) between GIR and HC, with 72 remaining significant after FDR adjustment (*P*_*FDR*_ < 0.05). When PIR was compared with HC, 53 metabolites (21 increased and 32 decreased) were significantly altered (*P* < 0.05) (Fig. 3B; Table S5), and 30 metabolites (9 increased and 21 decreased) remained significant after FDR adjustment (*P*_*FDR*_ < 0.05). When PIR was compared with GIR, 18 metabolites (7 increased and 11 decreased) were significantly altered (*P* < 0.05) (Fig. 3C, Table S6), while none remained significant after FDR adjustment.

**Fig. 4:**
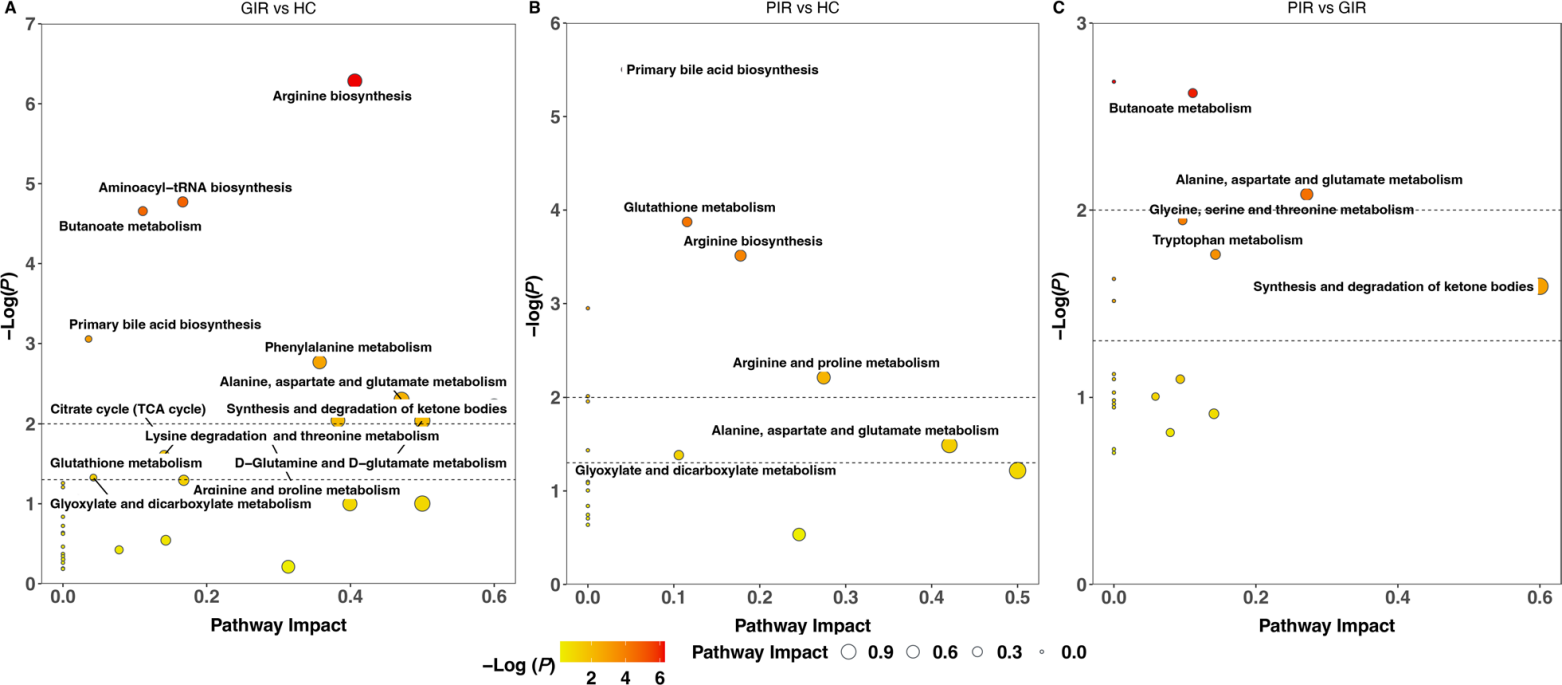
Metabolites pathway analysis.** A** Metabolites pathway analysis using the 88 significantly altered metabolites between HC and GIR. **B** Metabolites pathway analysis using the 53 significantly altered metabolites between HC and PIR. **C** Metabolites pathway analysis using the 18 significantly altered metabolites between GIR and PIR. HC, healthy controls; PIR, poor immunological responders; GIR, good immunological responders

Pathway analysis showed that differential metabolites between PIR and HC were enriched in primary bile acid biosynthesis, glutathione metabolism, arginine biosynthesis, arginine and proline metabolism, alanine, aspartate and glutamate metabolism, and glyoxylate and dicarboxylate metabolism (Fig. 4B). These metabolic pathways were also altered between HC and GIR (Fig. 4A). The significantly enriched pathways in PIR compared with GIR were included (1) butanoate metabolism, (2) alanine, aspartate, and glutamate metabolism, (3) glycine, serine, and threonine metabolism, (4) tryptophan metabolism, (5) synthesis and degradation of ketone bodies (Fig. 4C).

### Potential metabolites biomarkers related to immunological non-response

**Fig. 5:**
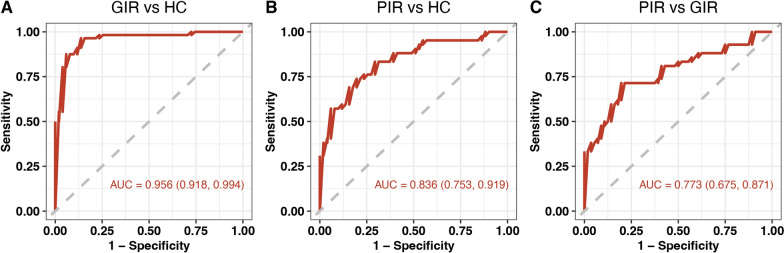
Metabolite markers for pairwise discriminations of HC, GIR, and PIR groups. **A** Receiver operating characteristic (ROC) analysis for the 11 metabolite markers discriminating GIR from HC. **B** ROC analysis for the 6 metabolite markers discriminating PIR from HC. **C** ROC analysis for the 4 metabolite markers discriminating PIR from GIR. HC, healthy controls; PIR, poor immunological responders; GIR, good immunological responders; AUC: area under curve

Using significantly altered metabolites (*P* < 0.05 for the comparison between PIR and GIR, *P*_*FDR*_ < 0.05 for the other two paired comparisons), we selected 11 metabolites as markers to classify GIR from HC using LASSO regression, including cinnamic acid, hydrocinnamic acid, azelaic acid, pyroglutamic acid, 3-hydroxybutyric acid, 3-hydroxyisovaleric acid, glutamic acid, adipic acid, phthalic acid, hyocholic acid and aspartic acid (Fig. 5A). PIR was classified from HC by 6 metabolite markers, including azelaic acid, pipecolic acid, pyroglutamic acid, cinnamic acid, hydrocinnamic acid, and indole-3-propionic acid (Fig. 5B). To distinguish PIR from GIR, 4 metabolites were identified, including dimethylglycine, cinnamic acid, 3-hydroxyisovaleric acid, and propionic acid (Fig. 5C). Screening of metabolite selection via LASSO regression is shown in the supplement (Figures S2–7).

### Immunologic response status discrimination with metabolites biomarkers

The area under the curve (AUC) was 0.956 [95% confidence interval (CI) 0.918–0.994] for the GIR-HC model (Fig. 5A), 0.836 (95% *CI*: 0.753–0.919) for the PIR-HC model (Fig. 5B), and 0.773 (95% *CI*: 0.675–0.871) for PIR-GIR model (Fig. 5C).

## Discussion

We observed significant differences in gut microbial metabolites among GIR, PIR, and HC. Specifically, we identified 88, 53, and 18 significantly altered metabolites when comparing GIR vs. HC, PIR vs. HC, and PIR vs. GIR, respectively. Furthermore, 11, 6, and 4 metabolites in each paired comparison were selected using LASSO regression. Our results showed that the selected metabolites can accurately differentiate different immunologic responses in HIV-infected MSM. These findings provide new insights into the metabolomic profiles of immunologic response among HIV-infected MSM and offer potential metabolites-related treatment strategies for PIR.

Metabolites have the potential to regulate immunologic responses among HIV-infected MSM. The decreasing trend of indole-3-propionic acid from HC to GIR to PIR was observed in our study. Tryptophan was lower in PIR compared with GIR. The tryptophan metabolism differed between PIR and GIR, indicating the biological relevance of the altered metabolisms. This finding is consistent with our previous study on gut microbiota composition in HIV patients. Our 16S rRNA sequencing results revealed that the abundance of *Bifidobacterium* was significantly higher in HC compared to both GIR and PIR [39]. *Bifidobacterium* is known to produce indole-3-lactic acid, a metabolite that plays a crucial role in regulating tryptophan metabolism [40]. This suggests a potential link between the reduced abundance of *Bifidobacterium* in HIV-infected MSM and alterations in tryptophan metabolism. A previous study also showed the level of indole-3-propionic acid was lower in PLHIV compared to HIV-uninfected individuals. However, no significant difference was observed between the two groups of PLHIV who experienced different increases in CD4 + T cell counts in the first 2 years after ART initiation [24]. Additionally, tryptophan was proven to increase CD4 + T cell levels in HIV-infected patients after ART initiation [41–43]. Another cohort study also reported metabolites associated with tryptophan metabolism in both HIV-infected and uninfected participants [44]. Indole-3-propionic acid, a product of tryptophan metabolism, shows anti-inflammatory properties [45]. Moreover, it plays a crucial role in preserving the integrity of mucosal barrier in the intestine [46]. Disruption of the gastrointestinal barrier is a hallmark of many chronic inflammatory diseases, including HIV infection [47]. A reduction in indole-3-propionic acid levels may reflect gut microbial dysbiosis and persistent inflammation in PLHIV [48, 49].

Importantly, we identified four novel metabolite markers related to immunologic response in HIV-infected MSM. Our study showed that propionic acid and cinnamic acid were depleted in PIR compared with GIR, which may imply the immunomodulatory properties. Consistent with this finding, our previous 16S rRNA sequencing data revealed that the relative abundance of Megamonas, a known producer of propionic acid, was significantly higher in GIR than in PIR [39]. A previous study reported elevated levels of propionic acid in PLHIV when compared to HIV-uninfected subjects [50]. Animal models have shown that propionic acid can significantly increase gut-associated Treg cells, subsequently reducing systemic immune responses and improving autoimmune disease symptoms [51]. A study on db/db mice has shown that cinnamic acid significantly increased the gene and protein expression of tryptophan metabolism while reducing serotonin levels, suggesting that cinnamic acid may regulate tryptophan metabolism [52]. This potential mechanism corroborates the observed changes in indole-3-propionic acid and tryptophan in this study. Furthermore, cinnamic acid was reported to significantly improve mitochondrial function. Evidence suggests that mitochondrial metabolism determines the differentiation, survival, and function of immune T cells. Targeting mitochondrial dysfunction was reported to restore the function of exhausted CD8 + T cells in HIV infection [53]. In contrast, the levels of dimethylglycine and 3-Hydroxyisovaleric acid were higher in PIR than in GIR. A study on HIV-infected women who received ART showed a depletion of dimethylglycine in participants with higher CD4 + T cell count [54]. A large prospective cohort study reported a positive linear relationship between dimethylglycine and C-reactive protein levels. This cohort study also suggested high plasma dimethylglycine levels may be related to the regulation of lipid and energy metabolism [55]. A pediatric HIV-infected cohort study also showed higher cerebrospinal fluid levels of 3-hydroxyisovaleric acid in HIV-infected individuals. This may be due to increased disruption of intestinal wall in PIR compared to GIR, resulting in malabsorption, which leads to a cascade of metabolic effects, including increased lactose levels, transketolase deficiency, and 3-methylcrotonyl-CoA carboxylase deficiency [56].

Therapeutic interventions targeting the aforementioned differential metabolites, including tryptophan-related products and the four metabolic biomarkers, may potentially reverse the presence or level of abnormal metabolites in PLHIV, thereby promoting immune response. Numerous experimental therapies targeting tryptophan metabolism are currently under investigation as novel cancer treatments. Epacadostat, a highly selective and orally bioavailable Indoleamine 2,3‐dioxygenase 1 inhibitor, is currently undergoing phase 3 clinical trials following positive results from multiple phases 1 and 2 clinical studies [57]. A large cohort study showed that after 2 weeks of multiple sclerosis immunotherapy supplemented with propionic acid, Treg cell levels significantly increase while Th1 and Th17 cell levels decreased [51]. However, further animal models and clinical studies are needed to evaluate whether these metabolites can effectively regulate the level of CD4 + T cell counts in PLHIV.

Our analysis also confirmed significant differences in butanoate metabolism, alanine, aspartate, and glutamate metabolism, and glycine, serine, and threonine metabolism between GIR and PIR. Butyrate plays a crucial role in the maintenance of the intestinal epithelial barrier, and mediating the effects of the gut microbiome on systemic and local immunity [58]. Previous evidence revealed a reduction of butyrate-producing gut microbiota in patients with inflammatory bowel diseases [59]. Studies showed that CD4 count was directly associated with alanine, and inversely correlated with glutamine concentrations in PLHIV [60]. Growing evidence shows glutamate receptors play an important role in T cell-mediated immunity and T cell development [61, 62]. As a substrate for the synthesis of purine and pyrimidine nucleotides, aspartate is crucial for the proliferation of lymphocytes [63]. Besides, aspartate enhances the intestinal immune responses by promoting the production of interleukin-1b in M1 macrophages [64]. Previous studies have also shown that glycine and serine metabolism is one of the most significantly associated metabolic pathways with HIV [24]. Glycine is necessary for facilitating the activity of glycine-gated chloride channels in leukocytes and macrophages. This activation has the potential to reduce cellular calcium levels, thereby inhibiting the production of cytokines and superoxide, and displaying anti-inflammatory properties [65].

Additionally, PIR in this study had lower CD4 + T and CD8 + T cell counts and were more likely to be classified as WHO stage IV. PLHIV with low CD4 + T cell counts or an AIDS-defining event, regardless of their CD4 + T cell count at diagnosis, are classified as late presenters. Late HIV diagnosis significantly reduces life expectancy while increasing healthcare costs, the risk of HIV-related comorbidities, and HIV transmission [66]. It is urgent to strengthen public health efforts to improve timely HIV diagnosis and treatment.

No significant differences in metabolite profiles were found between the two groups, regardless of whether rectal douching was performed in the previous 3 months. To date, no other studies have investigated the relationship between rectal douching and the gut microbiota or its metabolites. However, our study did not explore issues related to rectal douching behavior, which can vary in terms of frequency, types of devices, or types of fluid used. A systematic review indicated that rectal douching was associated with an increased risk of HIV infection among MSM [67]. More studies are needed to explore the relationship between rectal douching and health outcomes.

Our study had several notable strengths. Firstly, previous studies focused on exploring the differences in metabolites between HIV-infected and HIV-uninfected individuals, whereas the association of microbial metabolites and various immunologic responses in HIV-infected MSM has not been elucidated. Secondly, previous studies did not set sexual behavior as a screening criterion, while studies showed MSM have significantly different gut microbiota compared to non-MSM individuals [26, 68]. Our study is one of the first studies to compare microbial metabolites among HIV-infected MSM with poor immunological response, HIV-infected MSM with good immunological response, and HIV-uninfected MSM. Our study also includes healthy MSM as controls, with a large sample size and stringent inclusion criteria, ensuring more reliable results.

Several limitations should be considered. First, this is a cross-sectional study, which may not fully reflect the complex changes in the dynamic relationship between metabolites and immunologic response in HIV-infected MSM during ART. Longitudinal studies and mechanism experiments are needed to further explore the role of differential metabolites in the process of immunologic response. Second, we included only MSM from China and excluded individuals with a history of gastrointestinal surgery or special dietary habits, which may limit the generalizability of our study results. More studies are needed to explore altered gut microbiota metabolites that affect immune responses in non-MSM populations, such as women and heterosexual men, as well as to explore the potential impacts of special diets and gastrointestinal surgery. Third, potential confounding factors, including ART regimens, sexual behavior characteristics (such as frequency of sexual activity and number of sexual partners), co-infections, differences in recruitment sources between HC and HIV-infected MSM, recent food intake, alcohol consumption, and smoking status, may have influenced the metabolic profiles observed in this study. However, the comprehensive exclusion criteria, along with the similarity in gender, sexual orientation, and BMI across the three groups, the lack of significant differences in co-infection with HBV and HCV between the GIR and PIR groups, and the fact that most PLHIV received ART regimens including NRTIs, help mitigate the potential confounding effects of individual differences. Further studies are needed to reduce the influence of these confounding factors on the investigation of distinct gut microbial metabolites in PLHIV with varying immune responses. Fourth, missing values for demographics and clinical characteristics of HIV-infected and HIV-uninfected MSM were imputed with the median for continuous variables and the mode for categorical variables, which may not accurately reflect the actual situation. However, in this study, only a very small number of participants had missing values, so the impact on the results is minimal. Fifth, this study used targeted metabolomics profiling, which may not adequately reflect the altered metabolites among HC, GIR, and PIR. Lastly, further mechanistic studies and clinical trials are warranted for the validation of selected biomarkers.

## Conclusions

Our study proposed a novel perspective on the relationship between altered gut microbiota metabolites and immunologic response in HIV-infected MSM with suppressed viral load. Notably, dimethylglycine, cinnamic acid, 3-hydroxyisovaleric acid, and propionic acid were identified as key metabolites related to different immunologic responses. Furthermore, we demonstrate immunologic response is associated with indole-3-propionic acid and tryptophan, possibly attributed to the altered tryptophan metabolism. These findings highlight the potential of utilizing metabolites as biomarkers for disease progression after ART initiation in HIV-infected MSM and provide insight for future investigations into HIV treatment strategies. In addition, longitudinal studies and mechanism experiments are needed to further explore the role of differential metabolites in the process of immunologic response, establish causality, and determine their predictive value.

## Supplementary Information

Additional file 1.

Additional file 2.

## Data Availability

The datasets generated during and/or analyzed during the current study are not publicly available but are available from the corresponding author on reasonable request.

## Abbreviations

PLHIV: People living with HIV
ART: Antiretroviral therapy
MSM: Men who have sex with men
HC: Healthy controls
GIR: Good immunological responders
PIR: Poor immunological responders
AUC: Area under the curve
IQR: Interquartile range
PCA: Principal Component Analysis
PERMANOVA: Permutational multivariate analysis of variance
LASSO: Least absolute selection and shrinkage operator
ROC: Receiver operating characteristic
KEGG: Kyoto Encyclopedia of Genes and Genomes
*CI*: Confidence interval

## References

[1] Deeks SG, Lewin SR, Havlir DV. The end of AIDS: HIV infection as a chronic disease. Lancet. 2013;382(9903):1525–33. 24152939 10.1016/S0140-6736(13)61809-7 PMC4058441

[2] Yang X, Su B, Zhang X, Liu Y, Wu H, Zhang T. Incomplete immune reconstitution in HIV/AIDS patients on antiretroviral therapy: Challenges of immunological non-responders. J Leukoc Biol. 2020;107(4):597–612. 31965635 10.1002/JLB.4MR1019-189R PMC7187275

[3] Battegay M, Nüesch R, Hirschel B, Kaufmann GR. Immunological recovery and antiretroviral therapy in HIV-1 infection. Lancet Infect Dis. 2006;6(5):280–7. 16631548 10.1016/S1473-3099(06)70463-7

[4] Engsig FN, Zangerle R, Katsarou O, Dabis F, Reiss P, Gill J, et al. Long-term mortality in HIV-positive individuals virally suppressed for >3 years with incomplete CD4 recovery. Clin Infect Dis. 2014;58(9):1312–21. 24457342 10.1093/cid/ciu038 PMC6276895

[5] McHardy IH, Li X, Tong M, Ruegger P, Jacobs J, Borneman J, et al. HIV Infection is associated with compositional and functional shifts in the rectal mucosal microbiota. Microbiome. 2013;1(1):26. 24451087 10.1186/2049-2618-1-26 PMC3971626

[6] Vujkovic-Cvijin I, Dunham RM, Iwai S, Maher MC, Albright RG, Broadhurst MJ, et al. Dysbiosis of the gut microbiota is associated with HIV disease progression and tryptophan catabolism. Sci Transl Med. 2013;5(193): 193ra91. 23843452 10.1126/scitranslmed.3006438 PMC4094294

[7] Lee SC, Chua LL, Yap SH, Khang TF, Leng CY, Raja Azwa RI, et al. Enrichment of gut-derived Fusobacterium is associated with suboptimal immune recovery in HIV-infected individuals. Sci Rep. 2018;8(1):14277. 30250162 10.1038/s41598-018-32585-x PMC6155144

[8] Geng ST, Zhang ZY, Wang YX, Lu D, Yu J, Zhang JB, et al. Regulation of gut microbiota on immune reconstitution in patients with acquired immunodeficiency syndrome. Front Microbiol. 2020;11: 594820. 33193273 10.3389/fmicb.2020.594820 PMC7652894

[9] Serrano-Villar S, Ferrer M, Gosalbes MJ, Moreno S. How can the gut microbiota affect immune recovery in HIV-infected individuals? Future Microbiol. 2017;12:195–9. 28262047 10.2217/fmb-2016-0226

[10] Corrêa-Oliveira R, Fachi JL, Vieira A, Sato FT, Vinolo MA. Regulation of immune cell function by short-chain fatty acids. Clin Transl Immunol. 2016;5(4): e73. 10.1038/cti.2016.17 PMC4855267 27195116

[11] Li M, van Esch BCAM, Wagenaar GTM, Garssen J, Folkerts G, Henricks PAJ. Pro- and anti-inflammatory effects of short chain fatty acids on immune and endothelial cells. Eur J Pharmacol. 2018;831:52–9. 29750914 10.1016/j.ejphar.2018.05.003

[12] Babu H, Sperk M, Ambikan AT, Rachel G, Viswanathan VK, Tripathy SP, et al. Plasma metabolic signature and abnormalities in HIV-infected individuals on long-term successful antiretroviral therapy. Metabolites. 2019;9(10):210. 31574898 10.3390/metabo9100210 PMC6835959

[13] Peltenburg NC, Schoeman JC, Hou J, Mora F, Harms AC, Lowe SH, et al. Persistent metabolic changes in HIV-infected patients during the first year of combination antiretroviral therapy. Sci Rep. 2018;8(1):16947. 30446683 10.1038/s41598-018-35271-0 PMC6240055

[14] Lederman MM, Calabrese L, Funderburg NT, Clagett B, Medvik K, Bonilla H, et al. Immunologic failure despite suppressive antiretroviral therapy is related to activation and turnover of memory CD4 cells. J Infect Dis. 2011;204(8):1217–26. 21917895 10.1093/infdis/jir507 PMC3218674

[15] Teixeira L, Valdez H, McCune JM, Koup RA, Badley AD, Hellerstein MK, et al. Poor CD4 T cell restoration after suppression of HIV-1 replication may reflect lower thymic function. AIDS. 2001;15(14):1749–56. 11579235 10.1097/00002030-200109280-00002

[16] Dubé M, Tastet O, Dufour C, Sannier G, Brassard N, Delgado GG, et al. Spontaneous HIV expression during suppressive ART is associated with the magnitude and function of HIV-specific CD4(+) and CD8(+) T cells. Cell Host Microbe. 2023;31(9):1507-22.e5. 37708853 10.1016/j.chom.2023.08.006 PMC10542967

[17] Zhang L, Zheng J, Ahmed R, Huang G, Reid J, Mandal R, et al. A high-performing plasma metabolite panel for early-stage lung cancer detection. Cancers (Basel). 2020;12(3):622. 32156060 10.3390/cancers12030622 PMC7139410

[18] Ussher JR, Elmariah S, Gerszten RE, Dyck JR. The emerging role of metabolomics in the diagnosis and prognosis of cardiovascular disease. J Am Coll Cardiol. 2016;68(25):2850–70. 28007146 10.1016/j.jacc.2016.09.972

[19] Chen ZZ, Gerszten RE. Metabolomics and proteomics in type 2 diabetes. Circ Res. 2020;126(11):1613–27. 32437301 10.1161/CIRCRESAHA.120.315898 PMC11118076

[20] Beyrer C, Baral SD, van Griensven F, Goodreau SM, Chariyalertsak S, Wirtz AL, et al. Global epidemiology of HIV infection in men who have sex with men. Lancet. 2012;380(9839):367–77. 22819660 10.1016/S0140-6736(12)60821-6 PMC3805037

[21] Baral S, Sifakis F, Cleghorn F, Beyrer C. Elevated risk for HIV infection among men who have sex with men in low- and middle-income countries 2000–2006: a systematic review. PLoS Med. 2007;4(12): e339. 18052602 10.1371/journal.pmed.0040339 PMC2100144

[22] Vermund SH, Leigh-Brown AJ. The HIV epidemic: high-income countries. Cold Spring Harb Perspect Med. 2012;2(5): a007195. 22553497 10.1101/cshperspect.a007195 PMC3331688

[23] Serrano-Villar S, Rojo D, Martínez-Martínez M, Deusch S, Vázquez-Castellanos JF, Bargiela R, et al. Gut bacteria metabolism impacts immune recovery in HIV-infected individuals. EBioMedicine. 2016;8:203–16. 27428431 10.1016/j.ebiom.2016.04.033 PMC4919658

[24] Nyström S, Govender M, Yap SH, Kamarulzaman A, Rajasuriar R, Larsson M. HIV-infected individuals on ART with impaired immune recovery have altered plasma metabolite profiles. Open Forum Infect Dis. 2021;8(7): ofab288. 34258318 10.1093/ofid/ofab288 PMC8271132

[25] Lu L, Yang Y, Yang Z, Wu Y, Liu X, Li X, et al. Altered plasma metabolites and inflammatory networks in HIV-1 infected patients with different immunological responses after long-term antiretroviral therapy. Front Immunol. 2023;14:1254155. 37828979 10.3389/fimmu.2023.1254155 PMC10565217

[26] Noguera-Julian M, Rocafort M, Guillén Y, Rivera J, Casadellà M, Nowak P, et al. Gut microbiota linked to sexual preference and HIV infection. EBioMedicine. 2016;5:135–46. 27077120 10.1016/j.ebiom.2016.01.032 PMC4816837

[27] Tuddenham S, Koay WL, Sears C. HIV, sexual orientation, and gut microbiome interactions. Dig Dis Sci. 2020;65(3):800–17. 32030625 10.1007/s10620-020-06110-y PMC7301749

[28] Group AISaHCP. Consensus on diagnosis and management of immunological non-responders in HIV infection (Version 2023). Chin J Infect Dis 41.

[29] Rb-Silva R, Goios A, Kelly C, Teixeira P, João C, Horta A, et al. Definition of immunological nonresponse to antiretroviral therapy: a systematic review. J Acquir Immune Defic Syndr. 2019;82(5):452–61. 31592836 10.1097/QAI.0000000000002157

[30] Zhang J, Wang Z, Du W, Huang F, Jiang H, Bai J, et al. Twenty-five-year trends in dietary patterns among Chinese adults from 1991 to 2015. Nutrients. 2021;13(4):1327. 33923855 10.3390/nu13041327 PMC8072541

[31] Ross FC, Patangia D, Grimaud G, Lavelle A, Dempsey EM, Ross RP, et al. The interplay between diet and the gut microbiome: implications for health and disease. Nat Rev Microbiol. 2024;22:671–86. 39009882 10.1038/s41579-024-01068-4

[32] Xie G, Wang L, Chen T, Zhou K, Zhang Z, Li J, et al. A metabolite array technology for precision medicine. Anal Chem. 2021;93(14):5709–17. 33797874 10.1021/acs.analchem.0c04686

[33] Zhao L, Ni Y, Su M, Li H, Dong F, Chen W, et al. High throughput and quantitative measurement of microbial metabolome by gas chromatography/mass spectrometry using automated alkyl chloroformate derivatization. Anal Chem. 2017;89(10):5565–77. 28437060 10.1021/acs.analchem.7b00660 PMC5663283

[34] Tibshirani R. Regression shrinkage and selection via the lasso. J R Stat Soc: Ser B (Methodol). 1996;58(1):267–88.

[35] Yilmaz A, Ugur Z, Bisgin H, Akyol S, Bahado-Singh R, Wilson G, et al. Targeted metabolic profiling of urine highlights a potential biomarker panel for the diagnosis of alzheimer’s disease and mild cognitive impairment: a pilot study. Metabolites. 2020;10(9):357. 32878308 10.3390/metabo10090357 PMC7569858

[36] Atabaki-Pasdar N, Ohlsson M, Viñuela A, Frau F, Pomares-Millan H, Haid M, et al. Predicting and elucidating the etiology of fatty liver disease: a machine learning modeling and validation study in the IMI DIRECT cohorts. PLoS Med. 2020;17(6): e1003149. 32559194 10.1371/journal.pmed.1003149 PMC7304567

[37] Zhang P, Wang Z, Qiu H, Zhou W, Wang M, Cheng G. Machine learning applied to serum and cerebrospinal fluid metabolomes revealed altered arginine metabolism in neonatal sepsis with meningoencephalitis. Comput Struct Biotechnol J. 2021;19:3284–92. 34188777 10.1016/j.csbj.2021.05.024 PMC8207169

[38] Friedman J, Hastie T, Tibshirani R. Regularization paths for generalized linear models via coordinate descent. J Stat Softw. 2010;33(1):1–22. 20808728 PMC2929880

[39] Zhao H, Feng A, Luo D, Wu H, Zhang G, Zhang L, et al. Altered gut microbiota is associated with different immunologic responses to antiretroviral therapy in HIV-infected men who have sex with men. J Med Virol. 2023;95(3): e28674. 36920170 10.1002/jmv.28674

[40] Aragozzini F, Ferrari A, Pacini N, Gualandris R. Indole-3-lactic acid as a tryptophan metabolite produced by Bifidobacterium spp. Appl Environ Microbiol. 1979;38(3):544–6. 533277 10.1128/aem.38.3.544-546.1979 PMC243529

[41] Chen J, Shao J, Cai R, Shen Y, Zhang R, Liu L, et al. Anti-retroviral therapy decreases but does not normalize indoleamine 2,3-dioxygenase activity in HIV-infected patients. PLoS ONE. 2014;9(7): e100446. 24983463 10.1371/journal.pone.0100446 PMC4077698

[42] Neurauter G, Zangerle R, Widner B, Quirchmair G, Sarcletti M, Fuchs D. Effective antiretroviral therapy reduces degradation of tryptophan in patients with HIV-1 infection. Adv Exp Med Biol. 2003;527:317–23. 15206745 10.1007/978-1-4615-0135-0_35

[43] Jenabian MA, El-Far M, Vyboh K, Kema I, Costiniuk CT, Thomas R, et al. Immunosuppressive tryptophan catabolism and gut mucosal dysfunction following early HIV infection. J Infect Dis. 2015;212(3):355–66. 25616404 10.1093/infdis/jiv037

[44] Li SY, Yin LB, Ding HB, Liu M, Lv JN, Li JQ, et al. Altered lipid metabolites accelerate early dysfunction of T cells in HIV-infected rapid progressors by impairing mitochondrial function. Front Immunol. 2023;14:1106881. 36875092 10.3389/fimmu.2023.1106881 PMC9981933

[45] Konopelski P, Mogilnicka I. Biological effects of indole-3-propionic acid, a gut microbiota-derived metabolite, and its precursor tryptophan in mammals’ health and disease. Int J Mol Sci. 2022;23(3):1222. 35163143 10.3390/ijms23031222 PMC8835432

[46] Wlodarska M, Luo C, Kolde R, d’Hennezel E, Annand JW, Heim CE, et al. Indoleacrylic acid produced by commensal peptostreptococcus species suppresses inflammation. Cell Host Microbe. 2017;22(1):25-37.e6. 28704649 10.1016/j.chom.2017.06.007 PMC5672633

[47] Chung CY, Alden SL, Funderburg NT, Fu P, Levine AD. Progressive proximal-to-distal reduction in expression of the tight junction complex in colonic epithelium of virally-suppressed HIV+ individuals. PLoS Pathog. 2014;10(6): e1004198. 24968145 10.1371/journal.ppat.1004198 PMC4072797

[48] Rajasuriar R, Booth D, Solomon A, Chua K, Spelman T, Gouillou M, et al. Biological determinants of immune reconstitution in HIV-infected patients receiving antiretroviral therapy: the role of interleukin 7 and interleukin 7 receptor α and microbial translocation. J Infect Dis. 2010;202(8):1254–64. 20812848 10.1086/656369

[49] Rajasuriar R, Wright E, Lewin SR. Impact of antiretroviral therapy (ART) timing on chronic immune activation/inflammation and end-organ damage. Curr Opin HIV AIDS. 2015;10(1):35–42. 25415420 10.1097/COH.0000000000000118 PMC4301839

[50] González-Hernández LA, Ruiz-Briseño MDR, Sánchez-Reyes K, Alvarez-Zavala M, Vega-Magaña N, López-Iñiguez A, et al. Alterations in bacterial communities, SCFA and biomarkers in an elderly HIV-positive and HIV-negative population in western Mexico. BMC Infect Dis. 2019;19(1):234. 30845929 10.1186/s12879-019-3867-9 PMC6407185

[51] Duscha A, Gisevius B, Hirschberg S, Yissachar N, Stangl GI, Eilers E, et al. Propionic acid shapes the multiple sclerosis disease course by an immunomodulatory mechanism. Cell. 2020;180(6):1067-80.e16. 32160527 10.1016/j.cell.2020.02.035

[52] Wu Y, Wang MH, Yang T, Qin TY, Qin LL, Hu YM, et al. Mechanisms for improving hepatic glucolipid metabolism by cinnamic acid and cinnamic aldehyde: an insight provided by multi-omics. Front Nutr. 2021;8: 794841. 35087857 10.3389/fnut.2021.794841 PMC8786797

[53] Alrubayyi A, Moreno-Cubero E, Hameiri-Bowen D, Matthews R, Rowland-Jones S, Schurich A, et al. Functional restoration of exhausted CD8 T cells in chronic HIV-1 infection by targeting mitochondrial dysfunction. Front Immunol. 2022;13: 908697. 35865519 10.3389/fimmu.2022.908697 PMC9295450

[54] Mei Z, Yin MT, Sharma A, Wang Z, Peters BA, Chandran A, et al. Gut microbiota and plasma metabolites associated with bone mineral density in women with or at risk of HIV infection. AIDS. 2023;37(1):149–59. 36205320 10.1097/QAD.0000000000003400 PMC9742192

[55] Svingen GF, Ueland PM, Pedersen EK, Schartum-Hansen H, Seifert R, Ebbing M, et al. Plasma dimethylglycine and risk of incident acute myocardial infarction in patients with stable angina pectoris. Arterioscler Thromb Vasc Biol. 2013;33(8):2041–8. 23723367 10.1161/ATVBAHA.113.301714

[56] Thirion A, Loots DT, Williams ME, Solomons R, Mason S. An exploratory investigation of the CSF metabolic profile of HIV in a South African paediatric cohort using GCxGC-TOF/MS. Metabolomics. 2024;20(2):33. 38427142 10.1007/s11306-024-02098-y PMC10907482

[57] Sultana S, Elengickal A, Bensreti H, de Chantemèle EB, McGee-Lawrence ME, Hamrick MW. The kynurenine pathway in HIV, frailty and inflammaging. Front Immunol. 2023;14: 1244622. 37744363 10.3389/fimmu.2023.1244622 PMC10514395

[58] Siddiqui MT, Cresci GAM. The immunomodulatory functions of butyrate. J Inflamm Res. 2021;14:6025–41. 34819742 10.2147/JIR.S300989 PMC8608412

[59] Machiels K, Joossens M, Sabino J, De Preter V, Arijs I, Eeckhaut V, et al. A decrease of the butyrate-producing species Roseburia hominis and Faecalibacterium prausnitzii defines dysbiosis in patients with ulcerative colitis. Gut. 2014;63(8):1275–83. 24021287 10.1136/gutjnl-2013-304833

[60] McKnight TR, Yoshihara HA, Sitole LJ, Martin JN, Steffens F, Meyer D. A combined chemometric and quantitative NMR analysis of HIV/AIDS serum discloses metabolic alterations associated with disease status. Mol Biosyst. 2014;10(11):2889–97. 25105420 10.1039/c4mb00347k PMC4492936

[61] Boldyrev AA, Kazey VI, Leinsoo TA, Mashkina AP, Tyulina OV, Johnson P, et al. Rodent lymphocytes express functionally active glutamate receptors. Biochem Biophys Res Commun. 2004;324(1):133–9. 15464993 10.1016/j.bbrc.2004.09.019

[62] Rezzani R, Corsetti G, Rodella L, Angoscini P, Lonati C, Bianchi R. Cyclosporine-A treatment inhibits the expression of metabotropic glutamate receptors in rat thymus. Acta Histochem. 2003;105(1):81–7. 12666991 10.1078/0065-1281-00688

[63] Li P, Yin YL, Li D, Kim SW, Wu G. Amino acids and immune function. Br J Nutr. 2007;98(2):237–52. 17403271 10.1017/S000711450769936X

[64] Wang H, Zheng X, Liu B, Xia Y, Xin Z, Deng B, et al. Aspartate metabolism facilitates IL-1β production in inflammatory macrophages. Front Immunol. 2021;12: 753092. 34745126 10.3389/fimmu.2021.753092 PMC8567039

[65] Zhong Z, Wheeler MD, Li X, Froh M, Schemmer P, Yin M, et al. L-Glycine: a novel antiinflammatory, immunomodulatory, and cytoprotective agent. Curr Opin Clin Nutr Metab Care. 2003;6(2):229–40. 12589194 10.1097/00075197-200303000-00013

[66] Martin-Iguacel R, Reyes-Urueña J, Bruguera A, Aceitón J, Díaz Y, Moreno-Fornés S, et al. Determinants of long-term survival in late HIV presenters: the prospective PISCIS cohort study. EClinicalMedicine. 2022;52: 101600. 35958520 10.1016/j.eclinm.2022.101600 PMC9358427

[67] Li P, Yuan T, Fitzpatrick T, Smith K, Zhao J, Wu G, et al. Association between rectal douching and HIV and other sexually transmitted infections among men who have sex with men: a systematic review and meta-analysis. Sex Transm Infect. 2019;95(6):428–36. 31073094 10.1136/sextrans-2019-053964

[68] Kelley CF, Kraft CS, de Man TJ, Duphare C, Lee HW, Yang J, et al. The rectal mucosa and condomless receptive anal intercourse in HIV-negative MSM: implications for HIV transmission and prevention. Mucosal Immunol. 2017;10(4):996–1007. 27848950 10.1038/mi.2016.97 PMC5433931

