# Supplementary material for: Gut microbiota metabolites impact immunologic responses to antiretroviral therapy in HIV-infected men who have sex with men
Source: Infect Dis Poverty. 2025 Mar 18;14:21. doi: 10.1186/s40249-025-01291-y (PMC11917012; doi:10.1186/s40249-025-01291-y)
Supplement: Supplementary file 2 — Additional file 2. [file 40249_2025_1291_MOESM2_ESM.docx]

**Supplementary materials**

[Table S2 Infection characteristics and ART regimens of HIV-infected MSM 2](#_Toc188218616)

[Table S3. Differential metabolites among HC, GIR and PIR (nmol/g) 3](#_Toc188218617)

[Table S4. Differential metabolites between HC and GIR (nmol/g) 8](#_Toc188218618)

[Table S5. Differential metabolites between HC and PIR (nmol/g) 16](#_Toc188218619)

[Table S6. Differential metabolites between GIR and PIR (nmol/g) 24](#_Toc188218620)

[Figure S1 Principal component analysis (PCA) for MSM grouped by whether performed rectal douching in the previous 3 months 32](#_Toc188218621)

[Fig. S2. Identification of the optimal penalization coefficient (λ) (GIR vs HC) 33](#_Toc188218622)

[Fig. S3. LASSO coefficient profiles (GIR vs HC) 34](#_Toc188218623)

[Fig. S4. Identification of the optimal penalization coefficient (λ) (PIR vs HC) 35](#_Toc188218624)

[Fig. S5. LASSO coefficient profiles (PIR vs HC) 36](#_Toc188218625)

[Fig. S6. Identification of the optimal penalization coefficient (λ) (PIR vs GIR) 37](#_Toc188218626)

[Fig. S7. LASSO coefficient profiles (PIR vs GIR) 38](#_Toc188218627)

# Table S2 Infection characteristics and ART regimens of HIV-infected MSM

|  | **Overall** | **GIR** | **PIR** | **P value** |
| --- | --- | --- | --- | --- |
| **N** | 98 | 56 | 42 |  |
| **HBV infection, N (%)** | |  |  | 0.114 |
| Positive | 7 (7.1) | 2 (3.6) | 5 (11.9) |  |
| Negative | 77 (78.6) | 48 (85.7) | 29 (69.0) |  |
| Not reported | 14 (14.3) | 6 (10.7) | 8 (19.0) |  |
| **HCV infection, N (%)** | |  |  |  |
| Negative | 85 (86.7) | 51 (91.1) | 34 (81.0) | 0.246 |
| Not reported | 13 (13.3) | 5 (8.9) | 8 (19.0) |  |
| **Cytomegalovirus infection, N (%)** | | | | 0.01 |
| Negative | 89 (90.8) | 55 (98.2) | 34 (81.0) |  |
| Not reported | 9 (9.2) | 1 (1.8) | 8 (19.0) |  |
| **Baseline ART regimens, N (%)** | |  |  | <0.001 |
| NRTIs | 1 (1.0) | 0 (0.0) | 1 (2.4) |  |
| NRTIs+INSTIs | 6 (6.1) | 0 (0.0) | 6 (14.3) |  |
| NRTIs+NNRTIs | 83 (84.7) | 56 (100.0) | 27 (64.3) |  |
| NRTIs+PIs | 6 (6.1) | 0 (0.0) | 6 (14.3) |  |
| Not reported | 2 (2.0) | 0 (0.0) | 2 (4.8) |  |
| **Detailed baseline ART regimens, N (%)** | |  |  |  |
| 3TC+TDF | 1 (1.0) | 0 (0.0) | 1 (2.4) | 0.003 |
| DTG+3TC | 1 (1.0) | 0 (0.0) | 1 (2.4) |  |
| DTG+3TC+TDF | 4 (4.1) | 0 (0.0) | 4 (9.5) |  |
| DTG+FTC/TAF | 1 (1.0) | 0 (0.0) | 1 (2.4) |  |
| EFV+3TC+TDF | 80 (81.6) | 55 (98.2) | 25 (59.5) |  |
| EFV+AZT/3TC | 1 (1.0) | 1 (1.8) | 0 (0.0) |  |
| NVP+3TC+TDF | 1 (1.0) | 0 (0.0) | 1 (2.4) |  |
| NVP+AZT/3TC | 1 (1.0) | 0 (0.0) | 1 (2.4) |  |
| LPV/r+3TC | 2 (2.0) | 0 (0.0) | 2 (4.8) |  |
| LPV/r+3TC+TDF | 3 (3.1) | 0 (0.0) | 3 (7.1) |  |
| LPV/r+AZT/3TC | 1 (1.0) | 0 (0.0) | 1 (2.4) |  |
| Not reported | 2 (2.0) | 0 (0.0) | 2 (4.8) |  |

HBV, hepatitis B virus. HCV, hepatitis C virus

NRTIs, nucleoside reverse transcriptase inhibitors, including Zidovudine (AZT), Lamivudine (3TC), tenofovir disoproxil fumarate (TDF), tenofovir alafenamide (TAF), emtricitabine (FTC); NNRTIs, non-nucleoside reverse transcriptase inhibitors, including nevirapine (NVP), efavirenz (EFV); PIs, protease inhibitors, including lopinavir/ritonavir (LPV/r); INSTIs, integrase strand transfer inhibitors, including dolutegravir (DTG);

# Table S3. Differential metabolites among HC, GIR and PIR (nmol/g)

| **Metabolites** | **HC** | **GIR** | **PIR** | ***P*** **value** |
| --- | --- | --- | --- | --- |
| Cinnamic acid | 0.19 | 1.21 | 0.52 | 5.01E-11 |
| Isoallolithocholic acid | 0.27 | 0.07 | 0.06 | 7.87E-08 |
| Glycohyocholate | 0.02 | 0.20 | 0.04 | 7.27E-07 |
| Hydrocinnamic acid | 122.16 | 14.30 | 20.96 | 2.99E-06 |
| 2-Phenylpropionate | 50.24 | 6.02 | 8.61 | 3.59E-06 |
| Glutamic acid | 1160.62 | 603.05 | 684.06 | 5.83E-06 |
| Glycochenodeoxycholate | 1.26 | 6.46 | 4.12 | 5.94E-06 |
| Taurohyocholate | 0.02 | 0.36 | 0.04 | 6.40E-06 |
| Pyroglutamic acid | 1312.15 | 707.98 | 745.88 | 1.06E-05 |
| Adipic acid | 36.80 | 10.87 | 13.31 | 1.19E-05 |
| Glycoursodeoxycholic acid | 0.48 | 1.72 | 1.42 | 1.50E-05 |
| Aspartic acid | 686.21 | 323.99 | 484.74 | 1.53E-05 |
| Glycocholic acid | 1.04 | 5.09 | 6.05 | 1.98E-05 |
| Pipecolic acid | 17.30 | 7.55 | 9.02 | 2.13E-05 |
| gamma-Muricholic acid\hyocholic acid | 4.29 | 15.55 | 9.76 | 2.50E-05 |
| Methylglutaric acid | 3.03 | 0.86 | 1.28 | 3.18E-05 |
| Oxoadipic acid | 0.12 | 0.04 | 0.08 | 3.28E-05 |
| Acetoacetic acid | 195.68 | 72.74 | 121.31 | 4.19E-05 |
| Octanoic acid | 16.97 | 11.01 | 11.38 | 5.23E-05 |
| Azelaic acid | 12.87 | 6.62 | 7.95 | 6.53E-05 |
| Isolithocholic acid | 29.28 | 6.00 | 3.54 | 1.07E-04 |
| 3-Methyladipic acid | 3.43 | 1.71 | 1.83 | 1.57E-04 |
| Vanillic acid | 0.03 | 6.17 | 4.28 | 1.92E-04 |
| Glutaric acid | 83.74 | 38.72 | 37.83 | 2.55E-04 |
| 3,4-Dihydroxyhydrocinnamic acid | 6.32 | 84.95 | 36.33 | 2.77E-04 |
| Propionylcarnitine | 0.01 | 0.04 | 0.03 | 2.94E-04 |
| 7-Dehydrocholic acid | 9.26 | 46.40 | 61.42 | 3.08E-04 |
| 3-Hydroxyphenylacetic acid | 10.38 | 0.49 | 1.16 | 3.34E-04 |
| 3-(3-Hydroxyphenyl)-3-hydroxypropanoic acid | 2.06 | 22.90 | 9.96 | 3.58E-04 |
| Chenodeoxycholic acid | 79.79 | 536.61 | 407.61 | 3.78E-04 |
| Methylsuccinic acid | 46.10 | 22.92 | 19.49 | 4.21E-04 |
| Lithocholic acid | 433.72 | 113.62 | 82.08 | 6.87E-04 |
| Suberic acid | 7.02 | 4.14 | 4.42 | 9.46E-04 |
| Dimethylglycine | 2.86 | 2.19 | 2.93 | 1.02E-03 |
| Ursocholic acid | 18.03 | 80.91 | 144.58 | 1.16E-03 |
| Tauroursodeoxycholic acid | 0.09 | 0.69 | 0.34 | 1.17E-03 |
| Taurochenodeoxycholate | 1.08 | 7.93 | 2.18 | 1.31E-03 |
| Nicotinic acid | 223.37 | 117.86 | 157.49 | 1.49E-03 |
| omega-Muricholic acid | 1.93 | 4.99 | 4.38 | 1.54E-03 |
| Homovanillic acid | 5.31 | 47.58 | 21.88 | 1.74E-03 |
| Taurocholic acid | 1.11 | 7.38 | 3.89 | 1.82E-03 |
| Creatine | 24.24 | 266.99 | 82.63 | 2.09E-03 |
| Ethylmethylacetic acid | 1108.47 | 423.44 | 525.75 | 2.14E-03 |
| 3-Hydroxyisovaleric acid | 0.84 | 0.65 | 0.97 | 2.83E-03 |
| Tryptophan | 43.63 | 82.27 | 53.00 | 2.83E-03 |
| Butyric acid | 1205.76 | 966.68 | 973.44 | 3.14E-03 |
| Lysine | 889.22 | 546.91 | 639.82 | 3.20E-03 |
| beta-Muricholic acid | 13.12 | 48.31 | 49.42 | 3.48E-03 |
| Ornithine | 168.44 | 111.89 | 110.28 | 3.77E-03 |
| Cholic acid | 89.76 | 327.80 | 282.36 | 4.09E-03 |
| Glycolithocholic acid 3 sulfate | 0.49 | 1.40 | 0.52 | 4.09E-03 |
| Oxoglutaric acid | 10.79 | 3.81 | 8.98 | 4.20E-03 |
| Tauro omega-muricholate | 0.02 | 0.34 | 0.09 | 4.84E-03 |
| 23-Nordeoxycholic acid | 0.07 | 0.08 | 0.04 | 4.97E-03 |
| Decanoic acid | 3.96 | 2.56 | 2.70 | 5.24E-03 |
| Methionine | 118.61 | 72.74 | 116.54 | 6.11E-03 |
| Phthalic acid | 1.93 | 1.33 | 1.56 | 6.33E-03 |
| Ursodeoxycholic acid | 60.37 | 149.92 | 166.71 | 6.46E-03 |
| Indole-3-propionic acid | 11.39 | 8.16 | 6.50 | 9.39E-03 |
| Phenylacetic acid | 4166.13 | 1685.80 | 3370.08 | 1.03E-02 |
| Citramalic acid | 3.36 | 4.98 | 7.69 | 1.19E-02 |
| Isovaleric acid | 1423.67 | 814.48 | 934.72 | 1.48E-02 |
| Sarcosine | 16.82 | 25.30 | 18.01 | 1.79E-02 |
| Taurolithocholate | 0.16 | 0.21 | 0.04 | 2.05E-02 |
| 3-Dehydrocholic acid | 5.72 | 12.40 | 15.07 | 2.06E-02 |
| Picolinic acid | 0.14 | 0.07 | 0.09 | 2.07E-02 |
| Hippuric acid | 0.17 | 0.10 | 0.07 | 2.10E-02 |
| Citrulline | 185.99 | 133.66 | 141.71 | 2.52E-02 |
| Proline | 257.97 | 207.22 | 185.45 | 2.64E-02 |
| Propionic acid | 11273.30 | 12812.24 | 9574.06 | 2.80E-02 |
| 3beta-Ursodeoxycholic Acid | 5.03 | 11.02 | 13.63 | 3.04E-02 |
| Deoxycholic acid | 518.87 | 327.74 | 301.26 | 3.16E-02 |
| Citraconic acid | 0.21 | 0.13 | 0.18 | 3.21E-02 |
| Caproic acid | 50.57 | 32.62 | 20.16 | 3.66E-02 |
| Malic acid | 71.55 | 44.20 | 50.43 | 3.74E-02 |
| Phenyllactic acid | 1.18 | 3.00 | 3.52 | 3.74E-02 |
| alpha-Hyodeoxycholic acid | 7.54 | 3.92 | 4.10 | 3.83E-02 |
| Pentadecanoic acid | 60.43 | 38.60 | 30.97 | 4.04E-02 |
| Lithocholic acid 3 sulfate | 2.77 | 6.01 | 2.07 | 4.06E-02 |
| 3-Hydroxybutyric acid | 84.56 | 41.19 | 44.53 | 4.12E-02 |
| Mandelic acid | 0.25 | 0.18 | 0.25 | 4.58E-02 |
| alpha-Aminobutyric acid | 54.15 | 142.82 | 129.94 | 5.38E-02 |
| Heptanoic acid | 2.13 | 1.60 | 1.58 | 5.71E-02 |
| Glyceric acid | 35.94 | 44.86 | 27.78 | 6.78E-02 |
| Xylose | 1196.64 | 702.01 | 733.52 | 6.92E-02 |
| Glycine | 424.35 | 582.40 | 552.35 | 6.94E-02 |
| Phenylalanine | 40.15 | 147.99 | 111.24 | 7.03E-02 |
| Carnitine | 11.78 | 18.96 | 17.40 | 7.52E-02 |
| Arachidonic acid | 29.33 | 43.10 | 31.53 | 7.92E-02 |
| Serine | 215.91 | 151.56 | 164.13 | 8.70E-02 |
| Fumaric acid | 19.64 | 13.90 | 16.47 | 9.18E-02 |
| 2-Hydroxy-2-methylbutyric acid | 1.03 | 0.74 | 1.03 | 9.36E-02 |
| Glucose | 1839.26 | 2593.00 | 1640.20 | 1.02E-01 |
| Glycohyodeoxycholate | 0.03 | 0.06 | 0.04 | 1.03E-01 |
| Nor Cholic acid | 0.39 | 0.61 | 0.37 | 1.04E-01 |
| Taurodeoxycholate | 1.13 | 1.54 | 0.41 | 1.08E-01 |
| Dihomo-gamma-linolenic acid | 5.43 | 9.04 | 8.93 | 1.13E-01 |
| beta-Alanine | 84.97 | 187.59 | 128.81 | 1.20E-01 |
| Arginine | 50.58 | 28.78 | 60.70 | 1.21E-01 |
| ortho-Hydroxyphenylacetic acid | 0.39 | 0.28 | 0.33 | 1.23E-01 |
| Glutamine | 53.80 | 77.09 | 85.28 | 1.24E-01 |
| alpha-Hydroxyisobutyric acid | 2.05 | 1.28 | 1.48 | 1.26E-01 |
| Acetylcarnitine | 0.16 | 0.26 | 0.15 | 1.46E-01 |
| Ribonic acid | 2.97 | 6.23 | 5.14 | 1.47E-01 |
| Indolelactic acid | 0.89 | 1.08 | 0.97 | 1.56E-01 |
| Docosapentaenoic acid (22n-6) | 2.53 | 4.02 | 3.82 | 1.60E-01 |
| Valeric acid | 3588.09 | 3241.40 | 1994.09 | 1.60E-01 |
| Methylcysteine | 1.85 | 1.40 | 1.35 | 1.63E-01 |
| 2-Hydroxycinnamic acid | 0.10 | 0.07 | 0.01 | 1.63E-01 |
| Histidine | 84.61 | 82.04 | 69.67 | 1.65E-01 |
| 2-Phenylglycine | 0.24 | 0.19 | 0.19 | 1.66E-01 |
| Dodecanoic acid | 9.16 | 6.31 | 7.35 | 1.69E-01 |
| Indoleacrylic acid | 0.79 | 0.57 | 0.61 | 1.72E-01 |
| Imidazolepropionic acid | 25.05 | 62.29 | 38.08 | 1.78E-01 |
| Glycolithocholate | 0.34 | 0.26 | 0.14 | 1.82E-01 |
| 2-Butenoic acid | 17.79 | 14.71 | 12.18 | 1.84E-01 |
| 2-Hydroxy-3-methylbutyric acid | 8.29 | 10.77 | 11.32 | 1.87E-01 |
| Citric acid | 18.97 | 16.41 | 17.97 | 2.01E-01 |
| Myristic acid | 38.25 | 30.12 | 29.63 | 2.08E-01 |
| Docosahexaenoic acid | 13.18 | 19.13 | 16.39 | 2.08E-01 |
| Indoleacetic acid | 1.84 | 1.12 | 0.97 | 2.17E-01 |
| Itaconic acid | 0.45 | 0.20 | 0.34 | 2.19E-01 |
| Rhamnose | 72.96 | 99.58 | 85.13 | 2.23E-01 |
| Ribulose | 39.67 | 30.54 | 32.20 | 2.25E-01 |
| 3-Methyl-2-oxopentanoic acid | 5.85 | 4.03 | 5.46 | 2.48E-01 |
| Phenylpyruvic acid | 1.44 | 1.88 | 2.54 | 2.58E-01 |
| Eicosapentaenoic acid | 3.31 | 2.06 | 1.67 | 2.58E-01 |
| alpha-Ketoisovaleric acid | 6.44 | 6.70 | 7.56 | 2.77E-01 |
| Hydroxyphenyllactic acid | 6.42 | 10.68 | 9.20 | 2.80E-01 |
| Threonine | 130.21 | 111.58 | 104.01 | 2.84E-01 |
| Nonanoic acid | 3.73 | 3.64 | 3.59 | 2.89E-01 |
| alpha-Linolenic acid | 66.01 | 94.25 | 87.95 | 3.22E-01 |
| Alanine | 340.47 | 419.11 | 468.86 | 3.25E-01 |
| 4-Hydroxybenzoic acid | 5.34 | 7.75 | 6.42 | 3.29E-01 |
| 2-Hydroxycaproic acid | 3.02 | 2.74 | 2.23 | 3.32E-01 |
| Tartaric acid | 0.12 | 0.12 | 0.12 | 3.55E-01 |
| Malonic acid | 5.45 | 6.17 | 5.58 | 3.56E-01 |
| Docosapentaenoic acid | 10.30 | 12.45 | 9.97 | 3.58E-01 |
| Acetic acid | 34428.29 | 32721.86 | 33663.07 | 3.67E-01 |
| Xylulose | 27.97 | 22.71 | 24.52 | 3.82E-01 |
| Ketoleucine | 7.17 | 6.21 | 8.68 | 3.99E-01 |
| 3-Hydroxyhippuric acid | 0.90 | 1.29 | 0.33 | 4.19E-01 |
| Aminocaproic acid | 1.41 | 1.25 | 1.06 | 4.23E-01 |
| Aconitic acid | 8.63 | 8.02 | 9.37 | 4.68E-01 |
| N-Acetyl-D-glucosamine | 116.82 | 87.05 | 110.44 | 4.78E-01 |
| Glycodeoxycholic acid | 1.21 | 1.54 | 0.64 | 5.03E-01 |
| Benzoic acid | 8.04 | 8.30 | 7.07 | 5.10E-01 |
| Oleic acid | 1095.34 | 1224.26 | 1186.41 | 5.26E-01 |
| Tyrosine | 142.48 | 178.11 | 137.10 | 5.31E-01 |
| Adrenic acid | 5.97 | 7.51 | 5.22 | 5.31E-01 |
| Isocitric acid | 1.21 | 1.26 | 1.27 | 5.43E-01 |
| Palmitoleic acid | 62.02 | 76.87 | 82.36 | 5.51E-01 |
| Myristoleic acid | 1.44 | 1.38 | 1.36 | 5.86E-01 |
| Leucine | 145.95 | 195.72 | 254.66 | 5.95E-01 |
| Phenylacetylglutamine | 0.05 | 0.04 | 0.06 | 5.99E-01 |
| N-Phenylacetylphenylalanine | 0.07 | 0.06 | 0.08 | 6.05E-01 |
| Linoleic acid | 4104.22 | 4070.85 | 4863.93 | 6.31E-01 |
| 3-Aminosalicylic acid | 69.54 | 52.33 | 73.88 | 6.35E-01 |
| Fructose | 88.67 | 68.62 | 79.68 | 6.51E-01 |
| Oxalic acid | 10.44 | 9.40 | 9.12 | 6.75E-01 |
| p-Hydroxyphenylacetic acid | 58.52 | 53.66 | 68.87 | 6.88E-01 |
| gamma-Aminobutyric acid | 81.65 | 84.76 | 90.50 | 7.33E-01 |
| gamma-Linolenic acid | 3.89 | 4.81 | 4.63 | 7.46E-01 |
| Gluconolactone | 5.31 | 4.72 | 5.57 | 7.81E-01 |
| Hydroxypropionic acid | 107.97 | 120.15 | 97.54 | 7.94E-01 |
| Glycolic acid | 31.84 | 28.76 | 30.93 | 8.02E-01 |
| Valine | 202.09 | 251.66 | 319.11 | 8.19E-01 |
| Methylmalonic acid | 349.85 | 316.48 | 388.60 | 8.19E-01 |
| Isoleucine | 55.61 | 53.69 | 63.71 | 8.38E-01 |
| Threonic acid | 0.13 | 0.21 | 0.17 | 8.40E-01 |
| Asparagine | 7.03 | 8.96 | 7.28 | 8.40E-01 |
| Lactic acid | 61.68 | 69.48 | 65.65 | 8.80E-01 |
| Succinic acid | 572.69 | 486.57 | 496.04 | 9.20E-01 |
| 2-Hydroxybutyric acid | 7.08 | 6.32 | 5.91 | 9.32E-01 |

Metabolites highlighted in red are the differential metabolites identified among HC, GIR, and PIR. Differential metabolites were assessed by Kruskal-Wallis tests. HC, healthy controls; PIR, poor immunological responders; GIR, good immunological responders.

# Table S4. Differential metabolites between HC and GIR (nmol/g)

| **Metabolites** | **HC** | **GIR** | ***P value*** | ***FDR-P value*** | **FC** | **log2(FC)** |
| --- | --- | --- | --- | --- | --- | --- |
| Cinnamic acid | 0.19 | 1.21 | 6.85E-13 | 1.19E-10 | 6.47 | 2.69 |
| Glycohyocholate | 0.02 | 0.20 | 1.12E-07 | 9.78E-06 | 11.45 | 3.52 |
| Isoallolithocholic acid | 0.27 | 0.07 | 2.11E-07 | 1.22E-05 | 0.25 | -1.99 |
| Glutamic acid | 1160.62 | 603.05 | 1.02E-06 | 4.45E-05 | 0.52 | -0.94 |
| Hydrocinnamic acid | 122.16 | 14.30 | 1.86E-06 | 4.45E-05 | 0.12 | -3.09 |
| gamma-Muricholic acid\hyocholic acid | 4.29 | 15.55 | 1.91E-06 | 4.45E-05 | 3.62 | 1.86 |
| Taurohyocholate | 0.02 | 0.36 | 1.94E-06 | 4.45E-05 | 15.07 | 3.91 |
| Pyroglutamic acid | 1312.15 | 707.98 | 2.17E-06 | 4.45E-05 | 0.54 | -0.89 |
| 2-Phenylpropionate | 50.24 | 6.02 | 2.30E-06 | 4.45E-05 | 0.12 | -3.06 |
| Glycochenodeoxycholate | 1.26 | 6.46 | 2.68E-06 | 4.67E-05 | 5.14 | 2.36 |
| Adipic acid | 36.80 | 10.87 | 3.86E-06 | 6.11E-05 | 0.30 | -1.76 |
| Methylglutaric acid | 3.03 | 0.86 | 5.69E-06 | 7.84E-05 | 0.28 | -1.81 |
| Aspartic acid | 686.21 | 323.99 | 5.86E-06 | 7.84E-05 | 0.47 | -1.08 |
| Octanoic acid | 16.97 | 11.01 | 9.35E-06 | 0.00011167 | 0.65 | -0.62 |
| Acetoacetic acid | 195.68 | 72.74 | 9.63E-06 | 0.00011167 | 0.37 | -1.43 |
| Oxoadipic acid | 0.12 | 0.04 | 1.11E-05 | 0.00012083 | 0.31 | -1.69 |
| Pipecolic acid | 17.30 | 7.55 | 1.40E-05 | 0.000143 | 0.44 | -1.20 |
| Glycoursodeoxycholic acid | 0.48 | 1.72 | 1.80E-05 | 0.00017414 | 3.60 | 1.85 |
| Glycocholic acid | 1.04 | 5.09 | 2.59E-05 | 0.0002369 | 4.88 | 2.29 |
| Chenodeoxycholic acid | 79.79 | 536.61 | 4.47E-05 | 0.00038773 | 6.73 | 2.75 |
| 3-Methyladipic acid | 3.43 | 1.71 | 4.70E-05 | 0.00038773 | 0.50 | -1.01 |
| 3-Hydroxyphenylacetic acid | 10.38 | 0.49 | 4.90E-05 | 0.00038773 | 0.05 | -4.40 |
| Azelaic acid | 12.87 | 6.62 | 5.67E-05 | 0.00042883 | 0.51 | -0.96 |
| 3,4-Dihydroxyhydrocinnamic acid | 6.32 | 84.95 | 7.18E-05 | 0.00051161 | 13.44 | 3.75 |
| Vanillic acid | 0.03 | 6.17 | 7.35E-05 | 0.00051161 | 210.32 | 7.72 |
| 3-(3-Hydroxyphenyl)-3-hydroxypropanoic acid | 2.06 | 22.90 | 7.98E-05 | 0.00052093 | 11.11 | 3.47 |
| Propionylcarnitine | 0.01 | 0.04 | 8.14E-05 | 0.00052093 | 3.92 | 1.97 |
| omega-Muricholic acid | 1.93 | 4.99 | 8.38E-05 | 0.00052093 | 2.59 | 1.37 |
| Isolithocholic acid | 29.28 | 6.00 | 0.00011153 | 0.00066918 | 0.21 | -2.29 |
| Glutaric acid | 83.74 | 38.72 | 0.000147392 | 0.00085487 | 0.46 | -1.11 |
| Dimethylglycine | 2.86 | 2.19 | 0.000167059 | 0.00093768 | 0.77 | -0.39 |
| 7-Dehydrocholic acid | 9.26 | 46.40 | 0.000203729 | 0.00110778 | 5.01 | 2.33 |
| Taurochenodeoxycholate | 1.08 | 7.93 | 0.000297269 | 0.0015397 | 7.34 | 2.88 |
| Tauroursodeoxycholic acid | 0.09 | 0.69 | 0.00030086 | 0.0015397 | 7.92 | 2.99 |
| Creatine | 24.24 | 266.99 | 0.000339211 | 0.00168636 | 11.02 | 3.46 |
| Nicotinic acid | 223.37 | 117.86 | 0.000355785 | 0.00171345 | 0.53 | -0.92 |
| Methylsuccinic acid | 46.10 | 22.92 | 0.000364354 | 0.00171345 | 0.50 | -1.01 |
| Homovanillic acid | 5.31 | 47.58 | 0.000410166 | 0.00187363 | 8.97 | 3.16 |
| Ethylmethylacetic acid | 1108.47 | 423.44 | 0.000419951 | 0.00187363 | 0.38 | -1.39 |
| Lysine | 889.22 | 546.91 | 0.00044018 | 0.00191478 | 0.62 | -0.70 |
| Taurocholic acid | 1.11 | 7.38 | 0.000542977 | 0.00230434 | 6.65 | 2.73 |
| Suberic acid | 7.02 | 4.14 | 0.000683201 | 0.0028304 | 0.59 | -0.76 |
| Lithocholic acid | 433.72 | 113.62 | 0.000818875 | 0.00331189 | 0.26 | -1.93 |
| 3-Hydroxyisovaleric acid | 0.84 | 0.65 | 0.00083749 | 0.00331189 | 0.77 | -0.37 |
| Tryptophan | 43.63 | 82.27 | 0.001046477 | 0.00404638 | 1.89 | 0.91 |
| Methionine | 118.61 | 72.74 | 0.001093678 | 0.00413696 | 0.61 | -0.71 |
| Tauro omega-muricholate | 0.02 | 0.34 | 0.001162123 | 0.00430233 | 17.96 | 4.17 |
| Cholic acid | 89.76 | 327.80 | 0.001247352 | 0.00452165 | 3.65 | 1.87 |
| Decanoic acid | 3.96 | 2.56 | 0.001390391 | 0.00493731 | 0.65 | -0.63 |
| Oxoglutaric acid | 10.79 | 3.81 | 0.001420755 | 0.00494423 | 0.35 | -1.50 |
| Butyric acid | 1205.76 | 966.68 | 0.001616145 | 0.00551391 | 0.80 | -0.32 |
| beta-Muricholic acid | 13.12 | 48.31 | 0.001759836 | 0.00588868 | 3.68 | 1.88 |
| Phthalic acid | 1.93 | 1.33 | 0.001875213 | 0.00610629 | 0.69 | -0.53 |
| Glycolithocholic acid 3 sulfate | 0.49 | 1.40 | 0.001895056 | 0.00610629 | 2.86 | 1.52 |
| Ursocholic acid | 18.03 | 80.91 | 0.002360251 | 0.00746698 | 4.49 | 2.17 |
| Phenylacetic acid | 4166.13 | 1685.80 | 0.002670859 | 0.00829874 | 0.40 | -1.31 |
| Ursodeoxycholic acid | 60.37 | 149.92 | 0.002839784 | 0.00866881 | 2.48 | 1.31 |
| Ornithine | 168.44 | 111.89 | 0.003018411 | 0.00905523 | 0.66 | -0.59 |
| Isovaleric acid | 1423.67 | 814.48 | 0.003764545 | 0.01110222 | 0.57 | -0.81 |
| Sarcosine | 16.82 | 25.30 | 0.003916982 | 0.01135925 | 1.50 | 0.59 |
| Picolinic acid | 0.14 | 0.07 | 0.005721406 | 0.01632008 | 0.49 | -1.03 |
| Citrulline | 185.99 | 133.66 | 0.007808803 | 0.02191503 | 0.72 | -0.48 |
| 3-Hydroxybutyric acid | 84.56 | 41.19 | 0.008562624 | 0.02364915 | 0.49 | -1.04 |
| Indole-3-propionic acid | 11.39 | 8.16 | 0.00955263 | 0.02597121 | 0.72 | -0.48 |
| Phenyllactic acid | 1.18 | 3.00 | 0.010268124 | 0.02748698 | 2.55 | 1.35 |
| Citraconic acid | 0.21 | 0.13 | 0.012487432 | 0.03292141 | 0.64 | -0.64 |
| Malic acid | 71.55 | 44.20 | 0.012709092 | 0.0330057 | 0.62 | -0.69 |
| Proline | 257.97 | 207.22 | 0.013162432 | 0.03368034 | 0.80 | -0.32 |
| 3beta-Ursodeoxycholic Acid | 5.03 | 11.02 | 0.016325578 | 0.04094776 | 2.19 | 1.13 |
| alpha-Aminobutyric acid | 54.15 | 142.82 | 0.016473236 | 0.04094776 | 2.64 | 1.40 |
| Fumaric acid | 19.64 | 13.90 | 0.016755878 | 0.0410637 | 0.71 | -0.50 |
| 3-Dehydrocholic acid | 5.72 | 12.40 | 0.018345778 | 0.04433563 | 2.17 | 1.12 |
| Xylose | 1196.64 | 702.01 | 0.022995367 | 0.05481088 | 0.59 | -0.77 |
| Pentadecanoic acid | 60.43 | 38.60 | 0.025757389 | 0.06056467 | 0.64 | -0.65 |
| Mandelic acid | 0.25 | 0.18 | 0.026809997 | 0.06187593 | 0.71 | -0.50 |
| Serine | 215.91 | 151.56 | 0.02702627 | 0.06187593 | 0.70 | -0.51 |
| Caproic acid | 50.57 | 32.62 | 0.02926106 | 0.06612239 | 0.65 | -0.63 |
| 2-Hydroxy-2-methylbutyric acid | 1.03 | 0.74 | 0.030909817 | 0.06863457 | 0.72 | -0.48 |
| Phenylalanine | 40.15 | 147.99 | 0.031161671 | 0.06863457 | 3.69 | 1.88 |
| Carnitine | 11.78 | 18.96 | 0.033167261 | 0.07124819 | 1.61 | 0.69 |
| Citramalic acid | 3.36 | 4.98 | 0.033167261 | 0.07124819 | 1.48 | 0.57 |
| Glucose | 1839.26 | 2593.00 | 0.033685585 | 0.07147917 | 1.41 | 0.50 |
| Heptanoic acid | 2.13 | 1.60 | 0.035828715 | 0.0751108 | 0.75 | -0.41 |
| Glycohyodeoxycholate | 0.03 | 0.06 | 0.042964742 | 0.08861654 | 1.91 | 0.93 |
| ortho-Hydroxyphenylacetic acid | 0.39 | 0.28 | 0.043289687 | 0.08861654 | 0.72 | -0.48 |
| Arachidonic acid | 29.33 | 43.10 | 0.046279938 | 0.09255988 | 1.47 | 0.56 |
| beta-Alanine | 84.97 | 187.59 | 0.046279938 | 0.09255988 | 2.21 | 1.14 |
| Dihomo-gamma-linolenic acid | 5.43 | 9.04 | 0.048368198 | 0.09563712 | 1.66 | 0.74 |
| Glycine | 424.35 | 582.40 | 0.050535038 | 0.09879884 | 1.37 | 0.46 |
| Acetylcarnitine | 0.16 | 0.26 | 0.053548977 | 0.10311495 | 1.64 | 0.72 |
| Hippuric acid | 0.17 | 0.10 | 0.054315156 | 0.10311495 | 0.60 | -0.73 |
| 23-Nordeoxycholic acid | 0.07 | 0.08 | 0.054712362 | 0.10311495 | 1.09 | 0.12 |
| Dodecanoic acid | 9.16 | 6.31 | 0.055113161 | 0.10311495 | 0.69 | -0.54 |
| Lithocholic acid 3 sulfate | 2.77 | 6.01 | 0.055902811 | 0.10347967 | 2.17 | 1.12 |
| Glyceric acid | 35.94 | 44.86 | 0.057528852 | 0.10536863 | 1.25 | 0.32 |
| Docosapentaenoic acid (22n-6) | 2.53 | 4.02 | 0.060031935 | 0.10844663 | 1.59 | 0.67 |
| 2-Phenylglycine | 0.24 | 0.19 | 0.060455881 | 0.10844663 | 0.78 | -0.35 |
| Glutamine | 53.80 | 77.09 | 0.063509222 | 0.11276127 | 1.43 | 0.52 |
| Deoxycholic acid | 518.87 | 327.74 | 0.074937167 | 0.13041547 | 0.63 | -0.66 |
| Indolelactic acid | 0.89 | 1.08 | 0.074951422 | 0.13041547 | 1.21 | 0.27 |
| Imidazolepropionic acid | 25.05 | 62.29 | 0.079120494 | 0.13630659 | 2.49 | 1.31 |
| Docosahexaenoic acid | 13.18 | 19.13 | 0.080191715 | 0.13679763 | 1.45 | 0.54 |
| Itaconic acid | 0.45 | 0.20 | 0.081003381 | 0.13684066 | 0.45 | -1.14 |
| Rhamnose | 72.96 | 99.58 | 0.083476247 | 0.13966218 | 1.36 | 0.45 |
| Propionic acid | 11273.30 | 12812.24 | 0.086868897 | 0.14395417 | 1.14 | 0.18 |
| alpha-Hydroxyisobutyric acid | 2.05 | 1.28 | 0.090364107 | 0.14833353 | 0.62 | -0.68 |
| alpha-Hyodeoxycholic acid | 7.54 | 3.92 | 0.10149691 | 0.16505105 | 0.52 | -0.94 |
| 2-Hydroxy-3-methylbutyric acid | 8.29 | 10.77 | 0.108247586 | 0.1727989 | 1.30 | 0.38 |
| Ribonic acid | 2.97 | 6.23 | 0.108247586 | 0.1727989 | 2.10 | 1.07 |
| Indoleacetic acid | 1.84 | 1.12 | 0.111016422 | 0.17560779 | 0.61 | -0.71 |
| Arginine | 50.58 | 28.78 | 0.112315606 | 0.1760623 | 0.57 | -0.81 |
| Indoleacrylic acid | 0.79 | 0.57 | 0.121238029 | 0.18835194 | 0.72 | -0.48 |
| Citric acid | 18.97 | 16.41 | 0.122650738 | 0.18886043 | 0.87 | -0.21 |
| Hydroxyphenyllactic acid | 6.42 | 10.68 | 0.131952328 | 0.19969974 | 1.66 | 0.73 |
| Myristic acid | 38.25 | 30.12 | 0.131985459 | 0.19969974 | 0.79 | -0.34 |
| Nonanoic acid | 3.73 | 3.64 | 0.135216475 | 0.20282471 | 0.97 | -0.04 |
| Malonic acid | 5.45 | 6.17 | 0.138508266 | 0.20598665 | 1.13 | 0.18 |
| 2-Butenoic acid | 17.79 | 14.71 | 0.143011749 | 0.21088173 | 0.83 | -0.27 |
| 4-Hydroxybenzoic acid | 5.34 | 7.75 | 0.147007689 | 0.21495242 | 1.45 | 0.54 |
| Ribulose | 39.67 | 30.54 | 0.150517031 | 0.2182497 | 0.77 | -0.38 |
| alpha-Linolenic acid | 66.01 | 94.25 | 0.155900303 | 0.22418721 | 1.43 | 0.51 |
| Docosapentaenoic acid | 10.30 | 12.45 | 0.15772682 | 0.22495464 | 1.21 | 0.27 |
| Methylcysteine | 1.85 | 1.40 | 0.16330365 | 0.23101492 | 0.76 | -0.40 |
| Threonine | 130.21 | 111.58 | 0.18502677 | 0.25963434 | 0.86 | -0.22 |
| Phenylpyruvic acid | 1.44 | 1.88 | 0.208817134 | 0.29067345 | 1.31 | 0.39 |
| Valeric acid | 3588.09 | 3241.40 | 0.229872028 | 0.31744232 | 0.90 | -0.15 |
| 3-Methyl-2-oxopentanoic acid | 5.85 | 4.03 | 0.239699109 | 0.32840665 | 0.69 | -0.54 |
| Oleic acid | 1095.34 | 1224.26 | 0.24726126 | 0.33612077 | 1.12 | 0.16 |
| N-Acetyl-D-glucosamine | 116.82 | 87.05 | 0.249818711 | 0.33696477 | 0.75 | -0.42 |
| Xylulose | 27.97 | 22.71 | 0.252394585 | 0.33782044 | 0.81 | -0.30 |
| Nor Cholic acid | 0.39 | 0.61 | 0.265546688 | 0.34741307 | 1.57 | 0.65 |
| Acetic acid | 34428.29 | 32721.86 | 0.265551371 | 0.34741307 | 0.95 | -0.07 |
| Histidine | 84.61 | 82.04 | 0.265551371 | 0.34741307 | 0.97 | -0.04 |
| Taurodeoxycholate | 1.13 | 1.54 | 0.268233719 | 0.34830349 | 1.36 | 0.45 |
| Aminocaproic acid | 1.41 | 1.25 | 0.270595842 | 0.34876797 | 0.89 | -0.18 |
| 2-Hydroxycinnamic acid | 0.10 | 0.07 | 0.28601239 | 0.36592762 | 0.65 | -0.61 |
| Adrenic acid | 5.97 | 7.51 | 0.296136221 | 0.37478301 | 1.26 | 0.33 |
| 2-Hydroxycaproic acid | 3.02 | 2.74 | 0.297241701 | 0.37478301 | 0.91 | -0.14 |
| Myristoleic acid | 1.44 | 1.38 | 0.312278747 | 0.39091009 | 0.96 | -0.06 |
| Palmitoleic acid | 62.02 | 76.87 | 0.332092914 | 0.41266154 | 1.24 | 0.31 |
| Fructose | 88.67 | 68.62 | 0.335211635 | 0.41266154 | 0.77 | -0.37 |
| Phenylacetylglutamine | 0.05 | 0.04 | 0.336769764 | 0.41266154 | 0.94 | -0.09 |
| Oxalic acid | 10.44 | 9.40 | 0.354318427 | 0.43112872 | 0.90 | -0.15 |
| Tyrosine | 142.48 | 178.11 | 0.37746104 | 0.45609876 | 1.25 | 0.32 |
| N-Phenylacetylphenylalanine | 0.07 | 0.06 | 0.404375799 | 0.48474924 | 0.84 | -0.25 |
| Glycodeoxycholic acid | 1.21 | 1.54 | 0.406743616 | 0.48474924 | 1.28 | 0.35 |
| Linoleic acid | 4104.22 | 4070.85 | 0.422843051 | 0.5005081 | 0.99 | -0.01 |
| 3-Aminosalicylic acid | 69.54 | 52.33 | 0.437424763 | 0.51426965 | 0.75 | -0.41 |
| Tartaric acid | 0.12 | 0.12 | 0.456050391 | 0.53256891 | 0.95 | -0.07 |
| Taurolithocholate | 0.16 | 0.21 | 0.473152315 | 0.54885669 | 1.29 | 0.36 |
| p-Hydroxyphenylacetic acid | 58.52 | 53.66 | 0.482865578 | 0.55641464 | 0.92 | -0.13 |
| Alanine | 340.47 | 419.11 | 0.502529931 | 0.57526453 | 1.23 | 0.30 |
| Ketoleucine | 7.17 | 6.21 | 0.518561094 | 0.58938787 | 0.87 | -0.21 |
| Threonic acid | 0.13 | 0.21 | 0.52164214 | 0.58938787 | 1.69 | 0.76 |
| Glycolic acid | 31.84 | 28.76 | 0.534852539 | 0.59765423 | 0.90 | -0.15 |
| Methylmalonic acid | 349.85 | 316.48 | 0.538965366 | 0.59765423 | 0.90 | -0.14 |
| gamma-Linolenic acid | 3.89 | 4.81 | 0.539262727 | 0.59765423 | 1.24 | 0.31 |
| Glycolithocholate | 0.34 | 0.26 | 0.570306862 | 0.62643043 | 0.76 | -0.40 |
| Lactic acid | 61.68 | 69.48 | 0.572427805 | 0.62643043 | 1.13 | 0.17 |
| 3-Hydroxyhippuric acid | 0.90 | 1.29 | 0.583117705 | 0.6341405 | 1.43 | 0.51 |
| Hydroxypropionic acid | 107.97 | 120.15 | 0.593830879 | 0.63852134 | 1.11 | 0.15 |
| Gluconolactone | 5.31 | 4.72 | 0.598155045 | 0.63852134 | 0.89 | -0.17 |
| Valine | 202.09 | 251.66 | 0.598155045 | 0.63852134 | 1.25 | 0.32 |
| Isoleucine | 55.61 | 53.69 | 0.611212487 | 0.64848154 | 0.97 | -0.05 |
| Leucine | 145.95 | 195.72 | 0.669191412 | 0.70569276 | 1.34 | 0.42 |
| Asparagine | 7.03 | 8.96 | 0.682872879 | 0.71578242 | 1.27 | 0.35 |
| gamma-Aminobutyric acid | 81.65 | 84.76 | 0.805391912 | 0.83415591 | 1.04 | 0.05 |
| Succinic acid | 572.69 | 486.57 | 0.805391912 | 0.83415591 | 0.85 | -0.24 |
| 2-Hydroxybutyric acid | 7.08 | 6.32 | 0.863624112 | 0.88917512 | 0.89 | -0.16 |
| Benzoic acid | 8.04 | 8.30 | 0.913080736 | 0.93456499 | 1.03 | 0.05 |
| Aconitic acid | 8.63 | 8.02 | 0.932893968 | 0.94374174 | 0.93 | -0.11 |
| alpha-Ketoisovaleric acid | 6.44 | 6.70 | 0.932894132 | 0.94374174 | 1.04 | 0.06 |
| Eicosapentaenoic acid | 3.31 | 2.06 | 0.942817221 | 0.94826703 | 0.62 | -0.69 |
| Isocitric acid | 1.21 | 1.26 | 0.952746882 | 0.95274688 | 1.04 | 0.06 |

Metabolites highlighted in red are the differential metabolites identified between HC and GIR. Differential metabolites were assessed by Wilcoxon rank-sum tests between HC and GIR, FDR was used to correct for comparison. HC, healthy controls; GIR, good immunological responders.

# Table S5. Differential metabolites between HC and PIR (nmol/g)

| **Metabolites** | **HC** | **PIR** | ***P value*** | ***FDR-P value*** | **FC** | **log2(FC)** |
| --- | --- | --- | --- | --- | --- | --- |
| Isoallolithocholic acid | 0.27 | 0.06 | 4.20E-06 | 7.31E-04 | 0.21 | -2.24 |
| Glycoursodeoxycholic acid | 0.48 | 1.42 | 1.57E-04 | 9.21E-03 | 2.96 | 1.56 |
| Glycocholic acid | 1.04 | 6.05 | 1.68E-04 | 9.21E-03 | 5.79 | 2.53 |
| Hydrocinnamic acid | 122.16 | 20.96 | 2.57E-04 | 9.21E-03 | 0.17 | -2.54 |
| 2-Phenylpropionate | 50.24 | 8.61 | 2.65E-04 | 9.21E-03 | 0.17 | -2.55 |
| Glycochenodeoxycholate | 1.26 | 4.12 | 4.13E-04 | 1.13E-02 | 3.27 | 1.71 |
| Pipecolic acid | 17.30 | 9.02 | 4.77E-04 | 1.13E-02 | 0.52 | -0.94 |
| Azelaic acid | 12.87 | 7.95 | 5.20E-04 | 1.13E-02 | 0.62 | -0.69 |
| Isolithocholic acid | 29.28 | 3.54 | 6.00E-04 | 1.16E-02 | 0.12 | -3.05 |
| 23-Nordeoxycholic acid | 0.07 | 0.04 | 1.27E-03 | 1.90E-02 | 0.54 | -0.89 |
| Methylsuccinic acid | 46.10 | 19.49 | 1.45E-03 | 1.90E-02 | 0.42 | -1.24 |
| Ursocholic acid | 18.03 | 144.58 | 1.49E-03 | 1.90E-02 | 8.02 | 3.00 |
| Adipic acid | 36.80 | 13.31 | 1.53E-03 | 1.90E-02 | 0.36 | -1.47 |
| 7-Dehydrocholic acid | 9.26 | 61.42 | 1.61E-03 | 1.90E-02 | 6.63 | 2.73 |
| Glutaric acid | 83.74 | 37.83 | 1.70E-03 | 1.90E-02 | 0.45 | -1.15 |
| Lithocholic acid | 433.72 | 82.08 | 1.75E-03 | 1.90E-02 | 0.19 | -2.40 |
| Pyroglutamic acid | 1312.15 | 745.88 | 2.57E-03 | 2.55E-02 | 0.57 | -0.81 |
| Glutamic acid | 1160.62 | 684.06 | 2.64E-03 | 2.55E-02 | 0.59 | -0.76 |
| Suberic acid | 7.02 | 4.42 | 2.85E-03 | 2.61E-02 | 0.63 | -0.67 |
| Vanillic acid | 0.03 | 4.28 | 4.45E-03 | 3.77E-02 | 145.82 | 7.19 |
| 3-Methyladipic acid | 3.43 | 1.83 | 4.55E-03 | 3.77E-02 | 0.53 | -0.90 |
| Citramalic acid | 3.36 | 7.69 | 4.89E-03 | 3.87E-02 | 2.29 | 1.19 |
| Ornithine | 168.44 | 110.28 | 5.78E-03 | 4.37E-02 | 0.65 | -0.61 |
| Hippuric acid | 0.17 | 0.07 | 7.22E-03 | 4.97E-02 | 0.41 | -1.28 |
| Octanoic acid | 16.97 | 11.38 | 7.30E-03 | 4.97E-02 | 0.67 | -0.58 |
| Indole-3-propionic acid | 11.39 | 6.50 | 7.47E-03 | 4.97E-02 | 0.57 | -0.81 |
| Glycohyocholate | 0.02 | 0.04 | 7.71E-03 | 4.97E-02 | 2.43 | 1.28 |
| Aspartic acid | 686.21 | 484.74 | 8.19E-03 | 4.97E-02 | 0.71 | -0.50 |
| alpha-Hyodeoxycholic acid | 7.54 | 4.10 | 8.55E-03 | 4.97E-02 | 0.54 | -0.88 |
| Cinnamic acid | 0.19 | 0.52 | 8.57E-03 | 4.97E-02 | 2.78 | 1.48 |
| Methylglutaric acid | 3.03 | 1.28 | 9.17E-03 | 5.15E-02 | 0.42 | -1.24 |
| beta-Muricholic acid | 13.12 | 49.42 | 9.60E-03 | 5.17E-02 | 3.77 | 1.91 |
| Butyric acid | 1205.76 | 973.44 | 9.81E-03 | 5.17E-02 | 0.81 | -0.31 |
| Deoxycholic acid | 518.87 | 301.26 | 1.14E-02 | 5.86E-02 | 0.58 | -0.78 |
| Propionylcarnitine | 0.01 | 0.03 | 1.29E-02 | 6.40E-02 | 2.69 | 1.43 |
| Ursodeoxycholic acid | 60.37 | 166.71 | 1.55E-02 | 7.50E-02 | 2.76 | 1.47 |
| 3-Dehydrocholic acid | 5.72 | 15.07 | 1.68E-02 | 7.69E-02 | 2.63 | 1.40 |
| Taurocholic acid | 1.11 | 3.89 | 1.71E-02 | 7.69E-02 | 3.51 | 1.81 |
| Tauroursodeoxycholic acid | 0.09 | 0.34 | 1.72E-02 | 7.69E-02 | 3.83 | 1.94 |
| 3-Hydroxyphenylacetic acid | 10.38 | 1.16 | 2.19E-02 | 9.54E-02 | 0.11 | -3.17 |
| Taurolithocholate | 0.16 | 0.04 | 2.29E-02 | 9.74E-02 | 0.25 | -2.01 |
| Caproic acid | 50.57 | 20.16 | 2.49E-02 | 1.03E-01 | 0.40 | -1.33 |
| 3,4-Dihydroxyhydrocinnamic acid | 6.32 | 36.33 | 2.65E-02 | 1.07E-01 | 5.75 | 2.52 |
| Taurochenodeoxycholate | 1.08 | 2.18 | 3.09E-02 | 1.22E-01 | 2.02 | 1.01 |
| 3-(3-Hydroxyphenyl)-3-hydroxypropanoic acid | 2.06 | 9.96 | 3.19E-02 | 1.22E-01 | 4.83 | 2.27 |
| Ethylmethylacetic acid | 1108.47 | 525.75 | 3.22E-02 | 1.22E-01 | 0.47 | -1.08 |
| Proline | 257.97 | 185.45 | 3.34E-02 | 1.24E-01 | 0.72 | -0.48 |
| 3beta-Ursodeoxycholic Acid | 5.03 | 13.63 | 3.42E-02 | 1.24E-01 | 2.71 | 1.44 |
| Pentadecanoic acid | 60.43 | 30.97 | 3.54E-02 | 1.26E-01 | 0.51 | -0.96 |
| Cholic acid | 89.76 | 282.36 | 3.61E-02 | 1.26E-01 | 3.15 | 1.65 |
| Decanoic acid | 3.96 | 2.70 | 4.04E-02 | 1.38E-01 | 0.68 | -0.55 |
| Glycine | 424.35 | 552.35 | 4.43E-02 | 1.46E-01 | 1.30 | 0.38 |
| Heptanoic acid | 2.13 | 1.58 | 4.43E-02 | 1.46E-01 | 0.74 | -0.44 |
| Nicotinic acid | 223.37 | 157.49 | 5.13E-02 | 1.62E-01 | 0.71 | -0.50 |
| Valeric acid | 3588.09 | 1994.09 | 5.13E-02 | 1.62E-01 | 0.56 | -0.85 |
| Homovanillic acid | 5.31 | 21.88 | 6.34E-02 | 1.90E-01 | 4.12 | 2.04 |
| Isovaleric acid | 1423.67 | 934.72 | 6.34E-02 | 1.90E-01 | 0.66 | -0.61 |
| 2-Hydroxycinnamic acid | 0.10 | 0.01 | 6.45E-02 | 1.90E-01 | 0.05 | -4.20 |
| Glycolithocholate | 0.34 | 0.14 | 6.62E-02 | 1.90E-01 | 0.40 | -1.31 |
| Chenodeoxycholic acid | 79.79 | 407.61 | 6.65E-02 | 1.90E-01 | 5.11 | 2.35 |
| Methylcysteine | 1.85 | 1.35 | 6.67E-02 | 1.90E-01 | 0.73 | -0.45 |
| alpha-Hydroxyisobutyric acid | 2.05 | 1.48 | 6.79E-02 | 1.90E-01 | 0.72 | -0.47 |
| Ribonic acid | 2.97 | 5.14 | 7.77E-02 | 2.15E-01 | 1.73 | 0.79 |
| Phenyllactic acid | 1.18 | 3.52 | 7.90E-02 | 2.15E-01 | 2.99 | 1.58 |
| Malic acid | 71.55 | 50.43 | 8.45E-02 | 2.26E-01 | 0.70 | -0.50 |
| Picolinic acid | 0.14 | 0.09 | 8.57E-02 | 2.26E-01 | 0.61 | -0.71 |
| Indoleacrylic acid | 0.79 | 0.61 | 9.23E-02 | 2.32E-01 | 0.77 | -0.37 |
| 2-Butenoic acid | 17.79 | 12.18 | 9.28E-02 | 2.32E-01 | 0.68 | -0.55 |
| Eicosapentaenoic acid | 3.31 | 1.67 | 9.31E-02 | 2.32E-01 | 0.51 | -0.98 |
| Phenylalanine | 40.15 | 111.24 | 9.31E-02 | 2.32E-01 | 2.77 | 1.47 |
| Citrulline | 185.99 | 141.71 | 9.93E-02 | 2.43E-01 | 0.76 | -0.39 |
| Carnitine | 11.78 | 17.40 | 1.03E-01 | 2.44E-01 | 1.48 | 0.56 |
| Glutamine | 53.80 | 85.28 | 1.03E-01 | 2.44E-01 | 1.59 | 0.66 |
| Phenylacetic acid | 4166.13 | 3370.08 | 1.06E-01 | 2.49E-01 | 0.81 | -0.31 |
| Glycohyodeoxycholate | 0.03 | 0.04 | 1.09E-01 | 2.53E-01 | 1.23 | 0.30 |
| Xylose | 1196.64 | 733.52 | 1.11E-01 | 2.54E-01 | 0.61 | -0.71 |
| Myristic acid | 38.25 | 29.63 | 1.22E-01 | 2.75E-01 | 0.77 | -0.37 |
| Dihomo-gamma-linolenic acid | 5.43 | 8.93 | 1.24E-01 | 2.76E-01 | 1.64 | 0.72 |
| 2-Hydroxy-3-methylbutyric acid | 8.29 | 11.32 | 1.27E-01 | 2.80E-01 | 1.37 | 0.45 |
| Lysine | 889.22 | 639.82 | 1.31E-01 | 2.82E-01 | 0.72 | -0.47 |
| beta-Alanine | 84.97 | 128.81 | 1.33E-01 | 2.82E-01 | 1.52 | 0.60 |
| alpha-Aminobutyric acid | 54.15 | 129.94 | 1.35E-01 | 2.82E-01 | 2.40 | 1.26 |
| Ribulose | 39.67 | 32.20 | 1.37E-01 | 2.82E-01 | 0.81 | -0.30 |
| Serine | 215.91 | 164.13 | 1.37E-01 | 2.82E-01 | 0.76 | -0.40 |
| Tauro omega-muricholate | 0.02 | 0.09 | 1.39E-01 | 2.82E-01 | 4.70 | 2.23 |
| Phenylpyruvic acid | 1.44 | 2.54 | 1.39E-01 | 2.82E-01 | 1.76 | 0.82 |
| Taurodeoxycholate | 1.13 | 0.41 | 1.43E-01 | 2.87E-01 | 0.36 | -1.46 |
| Oxoadipic acid | 0.12 | 0.08 | 1.46E-01 | 2.88E-01 | 0.63 | -0.67 |
| Acetoacetic acid | 195.68 | 121.31 | 1.50E-01 | 2.93E-01 | 0.62 | -0.69 |
| omega-Muricholic acid | 1.93 | 4.38 | 1.54E-01 | 2.97E-01 | 2.27 | 1.18 |
| alpha-Ketoisovaleric acid | 6.44 | 7.56 | 1.58E-01 | 3.02E-01 | 1.17 | 0.23 |
| Indoleacetic acid | 1.84 | 0.97 | 1.66E-01 | 3.10E-01 | 0.53 | -0.92 |
| Threonine | 130.21 | 104.01 | 1.66E-01 | 3.10E-01 | 0.80 | -0.32 |
| Alanine | 340.47 | 468.86 | 1.68E-01 | 3.11E-01 | 1.38 | 0.46 |
| Phthalic acid | 1.93 | 1.56 | 1.71E-01 | 3.12E-01 | 0.81 | -0.31 |
| Creatine | 24.24 | 82.63 | 1.75E-01 | 3.16E-01 | 3.41 | 1.77 |
| 2-Hydroxycaproic acid | 3.02 | 2.23 | 1.76E-01 | 3.16E-01 | 0.74 | -0.44 |
| Acetic acid | 34428.29 | 33663.07 | 1.86E-01 | 3.29E-01 | 0.98 | -0.03 |
| 3-Hydroxybutyric acid | 84.56 | 44.53 | 1.88E-01 | 3.31E-01 | 0.53 | -0.93 |
| Xylulose | 27.97 | 24.52 | 2.15E-01 | 3.75E-01 | 0.88 | -0.19 |
| Docosapentaenoic acid (22n-6) | 2.53 | 3.82 | 2.20E-01 | 3.78E-01 | 1.51 | 0.59 |
| Aminocaproic acid | 1.41 | 1.06 | 2.47E-01 | 4.21E-01 | 0.75 | -0.42 |
| Nonanoic acid | 3.73 | 3.59 | 2.52E-01 | 4.25E-01 | 0.96 | -0.06 |
| alpha-Linolenic acid | 66.01 | 87.95 | 2.58E-01 | 4.32E-01 | 1.33 | 0.41 |
| Dimethylglycine | 2.86 | 2.93 | 2.65E-01 | 4.36E-01 | 1.02 | 0.03 |
| Nor Cholic acid | 0.39 | 0.37 | 2.65E-01 | 4.36E-01 | 0.97 | -0.04 |
| Leucine | 145.95 | 254.66 | 3.03E-01 | 4.92E-01 | 1.74 | 0.80 |
| Benzoic acid | 8.04 | 7.07 | 3.14E-01 | 4.98E-01 | 0.88 | -0.19 |
| ortho-Hydroxyphenylacetic acid | 0.39 | 0.33 | 3.16E-01 | 4.98E-01 | 0.85 | -0.23 |
| 2-Phenylglycine | 0.24 | 0.19 | 3.17E-01 | 4.98E-01 | 0.76 | -0.39 |
| Imidazolepropionic acid | 25.05 | 38.08 | 3.17E-01 | 4.98E-01 | 1.52 | 0.60 |
| Isocitric acid | 1.21 | 1.27 | 3.21E-01 | 4.99E-01 | 1.04 | 0.06 |
| Oxoglutaric acid | 10.79 | 8.98 | 3.29E-01 | 5.02E-01 | 0.83 | -0.26 |
| Propionic acid | 11273.30 | 9574.06 | 3.29E-01 | 5.02E-01 | 0.85 | -0.24 |
| Dodecanoic acid | 9.16 | 7.35 | 3.52E-01 | 5.32E-01 | 0.80 | -0.32 |
| Rhamnose | 72.96 | 85.13 | 3.56E-01 | 5.32E-01 | 1.17 | 0.22 |
| Taurohyocholate | 0.02 | 0.04 | 3.58E-01 | 5.32E-01 | 1.66 | 0.73 |
| Palmitoleic acid | 62.02 | 82.36 | 3.68E-01 | 5.43E-01 | 1.33 | 0.41 |
| Histidine | 84.61 | 69.67 | 3.77E-01 | 5.51E-01 | 0.82 | -0.28 |
| Aconitic acid | 8.63 | 9.37 | 3.89E-01 | 5.61E-01 | 1.09 | 0.12 |
| Linoleic acid | 4104.22 | 4863.93 | 3.94E-01 | 5.61E-01 | 1.19 | 0.25 |
| Tryptophan | 43.63 | 53.00 | 3.94E-01 | 5.61E-01 | 1.21 | 0.28 |
| Tartaric acid | 0.12 | 0.12 | 4.00E-01 | 5.64E-01 | 0.96 | -0.07 |
| Docosahexaenoic acid | 13.18 | 16.39 | 4.02E-01 | 5.64E-01 | 1.24 | 0.31 |
| gamma-Muricholic acid\hyocholic acid | 4.29 | 9.76 | 4.42E-01 | 6.06E-01 | 2.27 | 1.18 |
| Ketoleucine | 7.17 | 8.68 | 4.42E-01 | 6.06E-01 | 1.21 | 0.28 |
| N-Acetyl-D-glucosamine | 116.82 | 110.44 | 4.42E-01 | 6.06E-01 | 0.95 | -0.08 |
| Docosapentaenoic acid | 10.30 | 9.97 | 4.52E-01 | 6.14E-01 | 0.97 | -0.05 |
| gamma-Aminobutyric acid | 81.65 | 90.50 | 4.66E-01 | 6.25E-01 | 1.11 | 0.15 |
| 3-Hydroxyhippuric acid | 0.90 | 0.33 | 4.67E-01 | 6.25E-01 | 0.37 | -1.44 |
| Glycolithocholic acid 3 sulfate | 0.49 | 0.52 | 4.80E-01 | 6.37E-01 | 1.07 | 0.10 |
| Phenylacetylglutamine | 0.05 | 0.06 | 4.87E-01 | 6.42E-01 | 1.24 | 0.31 |
| Citraconic acid | 0.21 | 0.18 | 4.92E-01 | 6.44E-01 | 0.87 | -0.20 |
| Indolelactic acid | 0.89 | 0.97 | 5.04E-01 | 6.55E-01 | 1.09 | 0.12 |
| Hydroxyphenyllactic acid | 6.42 | 9.20 | 5.14E-01 | 6.63E-01 | 1.43 | 0.52 |
| Glycodeoxycholic acid | 1.21 | 0.64 | 5.19E-01 | 6.63E-01 | 0.53 | -0.93 |
| Myristoleic acid | 1.44 | 1.36 | 5.22E-01 | 6.63E-01 | 0.95 | -0.08 |
| Methionine | 118.61 | 116.54 | 5.34E-01 | 6.74E-01 | 0.98 | -0.03 |
| Malonic acid | 5.45 | 5.58 | 5.45E-01 | 6.82E-01 | 1.03 | 0.04 |
| Arginine | 50.58 | 60.70 | 5.50E-01 | 6.83E-01 | 1.20 | 0.26 |
| Valine | 202.09 | 319.11 | 5.76E-01 | 7.10E-01 | 1.58 | 0.66 |
| Oxalic acid | 10.44 | 9.12 | 5.92E-01 | 7.25E-01 | 0.87 | -0.20 |
| Fumaric acid | 19.64 | 16.47 | 5.97E-01 | 7.26E-01 | 0.84 | -0.25 |
| Oleic acid | 1095.34 | 1186.41 | 6.02E-01 | 7.28E-01 | 1.08 | 0.12 |
| Lithocholic acid 3 sulfate | 2.77 | 2.07 | 6.24E-01 | 7.49E-01 | 0.75 | -0.42 |
| Glucose | 1839.26 | 1640.20 | 6.40E-01 | 7.63E-01 | 0.89 | -0.17 |
| Methylmalonic acid | 349.85 | 388.60 | 6.63E-01 | 7.84E-01 | 1.11 | 0.15 |
| 2-Hydroxy-2-methylbutyric acid | 1.03 | 1.03 | 6.68E-01 | 7.86E-01 | 1.00 | 0.00 |
| 3-Methyl-2-oxopentanoic acid | 5.85 | 5.46 | 6.80E-01 | 7.86E-01 | 0.93 | -0.10 |
| 4-Hydroxybenzoic acid | 5.34 | 6.42 | 6.80E-01 | 7.86E-01 | 1.20 | 0.27 |
| 2-Hydroxybutyric acid | 7.08 | 5.91 | 6.82E-01 | 7.86E-01 | 0.83 | -0.26 |
| Succinic acid | 572.69 | 496.04 | 6.91E-01 | 7.91E-01 | 0.87 | -0.21 |
| Sarcosine | 16.82 | 18.01 | 7.34E-01 | 8.33E-01 | 1.07 | 0.10 |
| Gluconolactone | 5.31 | 5.57 | 7.37E-01 | 8.33E-01 | 1.05 | 0.07 |
| Acetylcarnitine | 0.16 | 0.15 | 7.60E-01 | 8.54E-01 | 0.93 | -0.11 |
| Fructose | 88.67 | 79.68 | 7.66E-01 | 8.55E-01 | 0.90 | -0.15 |
| Hydroxypropionic acid | 107.97 | 97.54 | 7.72E-01 | 8.56E-01 | 0.90 | -0.15 |
| Lactic acid | 61.68 | 65.65 | 7.96E-01 | 8.77E-01 | 1.06 | 0.09 |
| Asparagine | 7.03 | 7.28 | 8.08E-01 | 8.84E-01 | 1.04 | 0.05 |
| Threonic acid | 0.13 | 0.17 | 8.24E-01 | 8.96E-01 | 1.34 | 0.42 |
| Itaconic acid | 0.45 | 0.34 | 8.56E-01 | 9.25E-01 | 0.76 | -0.40 |
| p-Hydroxyphenylacetic acid | 58.52 | 68.87 | 8.62E-01 | 9.26E-01 | 1.18 | 0.24 |
| Adrenic acid | 5.97 | 5.22 | 8.68E-01 | 9.27E-01 | 0.87 | -0.19 |
| Mandelic acid | 0.25 | 0.25 | 8.86E-01 | 9.40E-01 | 1.02 | 0.03 |
| 3-Hydroxyisovaleric acid | 0.84 | 0.97 | 8.99E-01 | 9.48E-01 | 1.16 | 0.21 |
| Arachidonic acid | 29.33 | 31.53 | 9.05E-01 | 9.48E-01 | 1.07 | 0.10 |
| Isoleucine | 55.61 | 63.71 | 9.29E-01 | 9.68E-01 | 1.15 | 0.20 |
| N-Phenylacetylphenylalanine | 0.07 | 0.08 | 9.38E-01 | 9.72E-01 | 1.08 | 0.11 |
| Glyceric acid | 35.94 | 27.78 | 9.48E-01 | 9.76E-01 | 0.77 | -0.37 |
| Citric acid | 18.97 | 17.97 | 9.54E-01 | 9.76E-01 | 0.95 | -0.08 |
| Tyrosine | 142.48 | 137.10 | 9.66E-01 | 9.83E-01 | 0.96 | -0.06 |
| Glycolic acid | 31.84 | 30.93 | 9.72E-01 | 9.84E-01 | 0.97 | -0.04 |
| gamma-Linolenic acid | 3.89 | 4.63 | 9.91E-01 | 9.96E-01 | 1.19 | 0.25 |
| 3-Aminosalicylic acid | 69.54 | 73.88 | 9.97E-01 | 9.97E-01 | 1.06 | 0.09 |

Metabolites highlighted in red are the differential metabolites identified between HC and PIR. Differential metabolites were assessed by Wilcoxon rank-sum tests between HC and PIR, FDR was used to correct for comparison. HC, healthy controls; PIR, poor immunological responders.

# Table S6. Differential metabolites between GIR and PIR (nmol/g)

| **Metabolites** | **GIR** | **PIR** | ***P value*** | ***FDR-P value*** | **FC** | **log2(FC)** |
| --- | --- | --- | --- | --- | --- | --- |
| Taurohyocholate | 0.36 | 0.04 | 1.07E-03 | 1.87E-01 | 0.11 | -3.18 |
| Cinnamic acid | 1.21 | 0.52 | 2.25E-03 | 1.96E-01 | 0.43 | -1.22 |
| Oxoadipic acid | 0.04 | 0.08 | 4.27E-03 | 2.48E-01 | 2.04 | 1.03 |
| Acetoacetic acid | 72.74 | 121.31 | 7.49E-03 | 2.63E-01 | 1.67 | 0.74 |
| gamma-Muricholic acid\hyocholic acid | 15.55 | 9.76 | 7.56E-03 | 2.63E-01 | 0.63 | -0.67 |
| Propionic acid | 12812.24 | 9574.06 | 9.65E-03 | 2.80E-01 | 0.75 | -0.42 |
| Taurolithocholate | 0.21 | 0.04 | 1.17E-02 | 2.91E-01 | 0.19 | -2.37 |
| 3-Hydroxyisovaleric acid | 0.65 | 0.97 | 1.70E-02 | 3.07E-01 | 1.50 | 0.59 |
| Glycolithocholic acid 3 sulfate | 1.40 | 0.52 | 1.77E-02 | 3.07E-01 | 0.38 | -1.41 |
| Lithocholic acid 3 sulfate | 6.01 | 2.07 | 1.95E-02 | 3.07E-01 | 0.34 | -1.54 |
| Tryptophan | 82.27 | 53.00 | 2.10E-02 | 3.07E-01 | 0.64 | -0.63 |
| Glycohyocholate | 0.20 | 0.04 | 2.12E-02 | 3.07E-01 | 0.21 | -2.23 |
| Aspartic acid | 323.99 | 484.74 | 3.21E-02 | 4.14E-01 | 1.50 | 0.58 |
| Oxoglutaric acid | 3.81 | 8.98 | 3.33E-02 | 4.14E-01 | 2.36 | 1.24 |
| Nor Cholic acid | 0.61 | 0.37 | 3.82E-02 | 4.14E-01 | 0.62 | -0.70 |
| Dimethylglycine | 2.19 | 2.93 | 3.97E-02 | 4.14E-01 | 1.34 | 0.42 |
| Glyceric acid | 44.86 | 27.78 | 4.04E-02 | 4.14E-01 | 0.62 | -0.69 |
| Mandelic acid | 0.18 | 0.25 | 4.64E-02 | 4.48E-01 | 1.44 | 0.53 |
| Methionine | 72.74 | 116.54 | 6.05E-02 | 5.20E-01 | 1.60 | 0.68 |
| Taurodeoxycholate | 1.54 | 0.41 | 6.10E-02 | 5.20E-01 | 0.27 | -1.91 |
| Histidine | 82.04 | 69.67 | 6.77E-02 | 5.20E-01 | 0.85 | -0.24 |
| Arginine | 28.78 | 60.70 | 6.87E-02 | 5.20E-01 | 2.11 | 1.08 |
| Arachidonic acid | 43.10 | 31.53 | 6.88E-02 | 5.20E-01 | 0.73 | -0.45 |
| Citraconic acid | 0.13 | 0.18 | 7.68E-02 | 5.43E-01 | 1.36 | 0.44 |
| Sarcosine | 25.30 | 18.01 | 7.80E-02 | 5.43E-01 | 0.71 | -0.49 |
| Phthalic acid | 1.33 | 1.56 | 8.43E-02 | 5.48E-01 | 1.17 | 0.23 |
| Creatine | 266.99 | 82.63 | 8.69E-02 | 5.48E-01 | 0.31 | -1.69 |
| 3,4-Dihydroxyhydrocinnamic acid | 84.95 | 36.33 | 8.82E-02 | 5.48E-01 | 0.43 | -1.23 |
| 3-(3-Hydroxyphenyl)-3-hydroxypropanoic acid | 22.90 | 9.96 | 1.04E-01 | 6.15E-01 | 0.43 | -1.20 |
| 3-Methyl-2-oxopentanoic acid | 4.03 | 5.46 | 1.12E-01 | 6.15E-01 | 1.35 | 0.44 |
| Tauro omega-muricholate | 0.34 | 0.09 | 1.13E-01 | 6.15E-01 | 0.26 | -1.93 |
| Chenodeoxycholic acid | 536.61 | 407.61 | 1.13E-01 | 6.15E-01 | 0.76 | -0.40 |
| Methylglutaric acid | 0.86 | 1.28 | 1.20E-01 | 6.33E-01 | 1.48 | 0.57 |
| Homovanillic acid | 47.58 | 21.88 | 1.29E-01 | 6.60E-01 | 0.46 | -1.12 |
| Citric acid | 16.41 | 17.97 | 1.34E-01 | 6.68E-01 | 1.10 | 0.13 |
| Nicotinic acid | 117.86 | 157.49 | 1.42E-01 | 6.77E-01 | 1.34 | 0.42 |
| 23-Nordeoxycholic acid | 0.08 | 0.04 | 1.44E-01 | 6.77E-01 | 0.50 | -1.01 |
| alpha-Ketoisovaleric acid | 6.70 | 7.56 | 1.60E-01 | 6.86E-01 | 1.13 | 0.17 |
| 2-Hydroxy-2-methylbutyric acid | 0.74 | 1.03 | 1.67E-01 | 6.86E-01 | 1.40 | 0.49 |
| Tartaric acid | 0.12 | 0.12 | 1.74E-01 | 6.86E-01 | 1.00 | 0.00 |
| Glutamic acid | 603.05 | 684.06 | 1.76E-01 | 6.86E-01 | 1.13 | 0.18 |
| Propionylcarnitine | 0.04 | 0.03 | 1.81E-01 | 6.86E-01 | 0.69 | -0.54 |
| Lysine | 546.91 | 639.82 | 1.81E-01 | 6.86E-01 | 1.17 | 0.23 |
| 3-Hydroxyhippuric acid | 1.29 | 0.33 | 1.82E-01 | 6.86E-01 | 0.26 | -1.95 |
| Glucose | 2593.00 | 1640.20 | 1.83E-01 | 6.86E-01 | 0.63 | -0.66 |
| Indolelactic acid | 1.08 | 0.97 | 1.85E-01 | 6.86E-01 | 0.90 | -0.15 |
| Ketoleucine | 6.21 | 8.68 | 1.85E-01 | 6.86E-01 | 1.40 | 0.48 |
| Acetylcarnitine | 0.26 | 0.15 | 1.98E-01 | 6.99E-01 | 0.56 | -0.83 |
| Vanillic acid | 6.17 | 4.28 | 1.98E-01 | 6.99E-01 | 0.69 | -0.53 |
| Aconitic acid | 8.02 | 9.37 | 2.05E-01 | 6.99E-01 | 1.17 | 0.23 |
| Glycolithocholate | 0.26 | 0.14 | 2.09E-01 | 6.99E-01 | 0.53 | -0.91 |
| Octanoic acid | 11.01 | 11.38 | 2.10E-01 | 6.99E-01 | 1.03 | 0.05 |
| Pyroglutamic acid | 707.98 | 745.88 | 2.13E-01 | 6.99E-01 | 1.05 | 0.08 |
| Phenylacetic acid | 1685.80 | 3370.08 | 2.18E-01 | 6.99E-01 | 2.00 | 1.00 |
| Adipic acid | 10.87 | 13.31 | 2.21E-01 | 6.99E-01 | 1.22 | 0.29 |
| Eicosapentaenoic acid | 2.06 | 1.67 | 2.26E-01 | 7.04E-01 | 0.81 | -0.30 |
| Taurochenodeoxycholate | 7.93 | 2.18 | 2.43E-01 | 7.43E-01 | 0.28 | -1.86 |
| 3-Hydroxyphenylacetic acid | 0.49 | 1.16 | 2.60E-01 | 7.68E-01 | 2.36 | 1.24 |
| Itaconic acid | 0.20 | 0.34 | 2.61E-01 | 7.68E-01 | 1.66 | 0.74 |
| omega-Muricholic acid | 4.99 | 4.38 | 2.68E-01 | 7.79E-01 | 0.88 | -0.19 |
| Alanine | 419.11 | 468.86 | 2.90E-01 | 7.88E-01 | 1.12 | 0.16 |
| Cholic acid | 327.80 | 282.36 | 2.96E-01 | 7.88E-01 | 0.86 | -0.22 |
| Tyrosine | 178.11 | 137.10 | 3.03E-01 | 7.88E-01 | 0.77 | -0.38 |
| Benzoic acid | 8.30 | 7.07 | 3.05E-01 | 7.88E-01 | 0.85 | -0.23 |
| Hydrocinnamic acid | 14.30 | 20.96 | 3.17E-01 | 7.88E-01 | 1.47 | 0.55 |
| Glycodeoxycholic acid | 1.54 | 0.64 | 3.20E-01 | 7.88E-01 | 0.41 | -1.28 |
| Hydroxyphenyllactic acid | 10.68 | 9.20 | 3.24E-01 | 7.88E-01 | 0.86 | -0.21 |
| 2-Phenylpropionate | 6.02 | 8.61 | 3.27E-01 | 7.88E-01 | 1.43 | 0.52 |
| 2-Hydroxycinnamic acid | 0.07 | 0.01 | 3.32E-01 | 7.88E-01 | 0.08 | -3.59 |
| Imidazolepropionic acid | 62.29 | 38.08 | 3.34E-01 | 7.88E-01 | 0.61 | -0.71 |
| Citramalic acid | 4.98 | 7.69 | 3.38E-01 | 7.88E-01 | 1.54 | 0.63 |
| Deoxycholic acid | 327.74 | 301.26 | 3.41E-01 | 7.88E-01 | 0.92 | -0.12 |
| Fumaric acid | 13.90 | 16.47 | 3.42E-01 | 7.88E-01 | 1.19 | 0.25 |
| ortho-Hydroxyphenylacetic acid | 0.28 | 0.33 | 3.42E-01 | 7.88E-01 | 1.19 | 0.25 |
| Isocitric acid | 1.26 | 1.27 | 3.43E-01 | 7.88E-01 | 1.00 | 0.00 |
| 4-Hydroxybenzoic acid | 7.75 | 6.42 | 3.45E-01 | 7.88E-01 | 0.83 | -0.27 |
| Ursocholic acid | 80.91 | 144.58 | 3.49E-01 | 7.88E-01 | 1.79 | 0.84 |
| Citrulline | 133.66 | 141.71 | 3.64E-01 | 8.05E-01 | 1.06 | 0.08 |
| Tauroursodeoxycholic acid | 0.69 | 0.34 | 3.75E-01 | 8.05E-01 | 0.48 | -1.05 |
| Hippuric acid | 0.10 | 0.07 | 3.77E-01 | 8.05E-01 | 0.69 | -0.54 |
| 3-Methyladipic acid | 1.71 | 1.83 | 3.79E-01 | 8.05E-01 | 1.07 | 0.10 |
| N-Phenylacetylphenylalanine | 0.06 | 0.08 | 3.87E-01 | 8.05E-01 | 1.29 | 0.36 |
| Decanoic acid | 2.56 | 2.70 | 3.91E-01 | 8.05E-01 | 1.05 | 0.08 |
| Lithocholic acid | 113.62 | 82.08 | 3.91E-01 | 8.05E-01 | 0.72 | -0.47 |
| Docosahexaenoic acid | 19.13 | 16.39 | 3.95E-01 | 8.05E-01 | 0.86 | -0.22 |
| 3-Aminosalicylic acid | 52.33 | 73.88 | 3.99E-01 | 8.05E-01 | 1.41 | 0.50 |
| Adrenic acid | 7.51 | 5.22 | 4.07E-01 | 8.05E-01 | 0.70 | -0.52 |
| Ethylmethylacetic acid | 423.44 | 525.75 | 4.07E-01 | 8.05E-01 | 1.24 | 0.31 |
| 2-Phenylglycine | 0.19 | 0.19 | 4.28E-01 | 8.35E-01 | 0.97 | -0.04 |
| Taurocholic acid | 7.38 | 3.89 | 4.32E-01 | 8.35E-01 | 0.53 | -0.92 |
| p-Hydroxyphenylacetic acid | 53.66 | 68.87 | 4.45E-01 | 8.50E-01 | 1.28 | 0.36 |
| Dodecanoic acid | 6.31 | 7.35 | 4.66E-01 | 8.72E-01 | 1.17 | 0.22 |
| Glycochenodeoxycholate | 6.46 | 4.12 | 4.66E-01 | 8.72E-01 | 0.64 | -0.65 |
| 3-Hydroxybutyric acid | 41.19 | 44.53 | 4.75E-01 | 8.76E-01 | 1.08 | 0.11 |
| Picolinic acid | 0.07 | 0.09 | 4.84E-01 | 8.76E-01 | 1.25 | 0.32 |
| gamma-Linolenic acid | 4.81 | 4.63 | 4.93E-01 | 8.76E-01 | 0.96 | -0.05 |
| Rhamnose | 99.58 | 85.13 | 5.02E-01 | 8.76E-01 | 0.85 | -0.23 |
| alpha-Hyodeoxycholic acid | 3.92 | 4.10 | 5.11E-01 | 8.76E-01 | 1.05 | 0.07 |
| alpha-Aminobutyric acid | 142.82 | 129.94 | 5.11E-01 | 8.76E-01 | 0.91 | -0.14 |
| 2-Hydroxycaproic acid | 2.74 | 2.23 | 5.18E-01 | 8.76E-01 | 0.81 | -0.30 |
| 3-Dehydrocholic acid | 12.40 | 15.07 | 5.37E-01 | 8.76E-01 | 1.22 | 0.28 |
| Docosapentaenoic acid | 12.45 | 9.97 | 5.39E-01 | 8.76E-01 | 0.80 | -0.32 |
| Isolithocholic acid | 6.00 | 3.54 | 5.39E-01 | 8.76E-01 | 0.59 | -0.76 |
| Malonic acid | 6.17 | 5.58 | 5.39E-01 | 8.76E-01 | 0.91 | -0.14 |
| Valeric acid | 3241.40 | 1994.09 | 5.44E-01 | 8.76E-01 | 0.62 | -0.70 |
| gamma-Aminobutyric acid | 84.76 | 90.50 | 5.49E-01 | 8.76E-01 | 1.07 | 0.09 |
| Pipecolic acid | 7.55 | 9.02 | 5.49E-01 | 8.76E-01 | 1.19 | 0.26 |
| Carnitine | 18.96 | 17.40 | 5.54E-01 | 8.76E-01 | 0.92 | -0.12 |
| Gluconolactone | 4.72 | 5.57 | 5.54E-01 | 8.76E-01 | 1.18 | 0.24 |
| Leucine | 195.72 | 254.66 | 5.54E-01 | 8.76E-01 | 1.30 | 0.38 |
| Isovaleric acid | 814.48 | 934.72 | 5.63E-01 | 8.82E-01 | 1.15 | 0.20 |
| Malic acid | 44.20 | 50.43 | 5.68E-01 | 8.82E-01 | 1.14 | 0.19 |
| Hydroxypropionic acid | 120.15 | 97.54 | 5.73E-01 | 8.82E-01 | 0.81 | -0.30 |
| Phenylpyruvic acid | 1.88 | 2.54 | 5.83E-01 | 8.89E-01 | 1.35 | 0.43 |
| Asparagine | 8.96 | 7.28 | 5.93E-01 | 8.89E-01 | 0.81 | -0.30 |
| Phenylalanine | 147.99 | 111.24 | 5.93E-01 | 8.89E-01 | 0.75 | -0.41 |
| Butyric acid | 966.68 | 973.44 | 6.18E-01 | 9.03E-01 | 1.01 | 0.01 |
| Docosapentaenoic acid (22n-6) | 4.02 | 3.82 | 6.23E-01 | 9.03E-01 | 0.95 | -0.08 |
| Glycolic acid | 28.76 | 30.93 | 6.23E-01 | 9.03E-01 | 1.08 | 0.10 |
| Isoleucine | 53.69 | 63.71 | 6.23E-01 | 9.03E-01 | 1.19 | 0.25 |
| N-Acetyl-D-glucosamine | 87.05 | 110.44 | 6.33E-01 | 9.10E-01 | 1.27 | 0.34 |
| Oleic acid | 1224.26 | 1186.41 | 6.43E-01 | 9.18E-01 | 0.97 | -0.05 |
| Fructose | 68.62 | 79.68 | 6.54E-01 | 9.25E-01 | 1.16 | 0.22 |
| Ribulose | 30.54 | 32.20 | 6.85E-01 | 9.57E-01 | 1.05 | 0.08 |
| Methylcysteine | 1.40 | 1.35 | 6.88E-01 | 9.57E-01 | 0.96 | -0.06 |
| Myristoleic acid | 1.38 | 1.36 | 7.47E-01 | 9.91E-01 | 0.99 | -0.02 |
| Pentadecanoic acid | 38.60 | 30.97 | 7.60E-01 | 9.91E-01 | 0.80 | -0.32 |
| 2-Butenoic acid | 14.71 | 12.18 | 7.76E-01 | 9.91E-01 | 0.83 | -0.27 |
| Phenylacetylglutamine | 0.04 | 0.06 | 7.85E-01 | 9.91E-01 | 1.32 | 0.40 |
| beta-Muricholic acid | 48.31 | 49.42 | 7.93E-01 | 9.91E-01 | 1.02 | 0.03 |
| Dihomo-gamma-linolenic acid | 9.04 | 8.93 | 7.93E-01 | 9.91E-01 | 0.99 | -0.02 |
| Nonanoic acid | 3.64 | 3.59 | 8.04E-01 | 9.91E-01 | 0.99 | -0.02 |
| Glutamine | 77.09 | 85.28 | 8.10E-01 | 9.91E-01 | 1.11 | 0.15 |
| Methylsuccinic acid | 22.92 | 19.49 | 8.10E-01 | 9.91E-01 | 0.85 | -0.23 |
| Ornithine | 111.89 | 110.28 | 8.10E-01 | 9.91E-01 | 0.99 | -0.02 |
| Threonine | 111.58 | 104.01 | 8.21E-01 | 9.91E-01 | 0.93 | -0.10 |
| 2-Hydroxy-3-methylbutyric acid | 10.77 | 11.32 | 8.27E-01 | 9.91E-01 | 1.05 | 0.07 |
| Xylose | 702.01 | 733.52 | 8.32E-01 | 9.91E-01 | 1.04 | 0.06 |
| Glycine | 582.40 | 552.35 | 8.38E-01 | 9.91E-01 | 0.95 | -0.08 |
| Threonic acid | 0.21 | 0.17 | 8.47E-01 | 9.91E-01 | 0.79 | -0.34 |
| Glycocholic acid | 5.09 | 6.05 | 8.55E-01 | 9.91E-01 | 1.19 | 0.25 |
| Azelaic acid | 6.62 | 7.95 | 8.60E-01 | 9.91E-01 | 1.20 | 0.27 |
| Caproic acid | 32.62 | 20.16 | 8.60E-01 | 9.91E-01 | 0.62 | -0.69 |
| Succinic acid | 486.57 | 496.04 | 8.60E-01 | 9.91E-01 | 1.02 | 0.03 |
| 3beta-Ursodeoxycholic Acid | 11.02 | 13.63 | 8.80E-01 | 9.91E-01 | 1.24 | 0.31 |
| 2-Hydroxybutyric acid | 6.32 | 5.91 | 8.83E-01 | 9.91E-01 | 0.94 | -0.10 |
| Glycohyodeoxycholate | 0.06 | 0.04 | 8.83E-01 | 9.91E-01 | 0.65 | -0.63 |
| Isoallolithocholic acid | 0.07 | 0.06 | 8.89E-01 | 9.91E-01 | 0.84 | -0.26 |
| Indoleacetic acid | 1.12 | 0.97 | 8.89E-01 | 9.91E-01 | 0.86 | -0.22 |
| Suberic acid | 4.14 | 4.42 | 8.89E-01 | 9.91E-01 | 1.07 | 0.09 |
| Ribonic acid | 6.23 | 5.14 | 8.94E-01 | 9.91E-01 | 0.83 | -0.28 |
| Glycoursodeoxycholic acid | 1.72 | 1.42 | 8.94E-01 | 9.91E-01 | 0.82 | -0.28 |
| Phenyllactic acid | 3.00 | 3.52 | 8.94E-01 | 9.91E-01 | 1.17 | 0.23 |
| beta-Alanine | 187.59 | 128.81 | 9.00E-01 | 9.91E-01 | 0.69 | -0.54 |
| Acetic acid | 32721.86 | 33663.07 | 9.06E-01 | 9.91E-01 | 1.03 | 0.04 |
| Serine | 151.56 | 164.13 | 9.06E-01 | 9.91E-01 | 1.08 | 0.11 |
| Xylulose | 22.71 | 24.52 | 9.06E-01 | 9.91E-01 | 1.08 | 0.11 |
| alpha-Linolenic acid | 94.25 | 87.95 | 9.34E-01 | 9.91E-01 | 0.93 | -0.10 |
| Methylmalonic acid | 316.48 | 388.60 | 9.46E-01 | 9.91E-01 | 1.23 | 0.30 |
| Indole-3-propionic acid | 8.16 | 6.50 | 9.51E-01 | 9.91E-01 | 0.80 | -0.33 |
| Indoleacrylic acid | 0.57 | 0.61 | 9.54E-01 | 9.91E-01 | 1.08 | 0.10 |
| Aminocaproic acid | 1.25 | 1.06 | 9.57E-01 | 9.91E-01 | 0.85 | -0.24 |
| alpha-Hydroxyisobutyric acid | 1.28 | 1.48 | 9.63E-01 | 9.91E-01 | 1.16 | 0.21 |
| Glutaric acid | 38.72 | 37.83 | 9.63E-01 | 9.91E-01 | 0.98 | -0.03 |
| 7-Dehydrocholic acid | 46.40 | 61.42 | 9.69E-01 | 9.91E-01 | 1.32 | 0.40 |
| Linoleic acid | 4070.85 | 4863.93 | 9.69E-01 | 9.91E-01 | 1.19 | 0.26 |
| Proline | 207.22 | 185.45 | 9.69E-01 | 9.91E-01 | 0.89 | -0.16 |
| Lactic acid | 69.48 | 65.65 | 9.74E-01 | 9.91E-01 | 0.94 | -0.08 |
| Heptanoic acid | 1.60 | 1.58 | 9.80E-01 | 9.91E-01 | 0.98 | -0.02 |
| Oxalic acid | 9.40 | 9.12 | 9.80E-01 | 9.91E-01 | 0.97 | -0.04 |
| Palmitoleic acid | 76.87 | 82.36 | 9.80E-01 | 9.91E-01 | 1.07 | 0.10 |
| Ursodeoxycholic acid | 149.92 | 166.71 | 9.80E-01 | 9.91E-01 | 1.11 | 0.15 |
| Myristic acid | 30.12 | 29.63 | 9.91E-01 | 9.97E-01 | 0.98 | -0.02 |
| Valine | 251.66 | 319.11 | 9.97E-01 | 9.97E-01 | 1.27 | 0.34 |

Metabolites highlighted in red are the differential metabolites identified between GIR and PIR. Differential metabolites were assessed by Wilcoxon rank-sum tests between PIR and GIR, FDR was used to correct for comparison. GIR, good immunological responders. PIR, poor immunological responders.


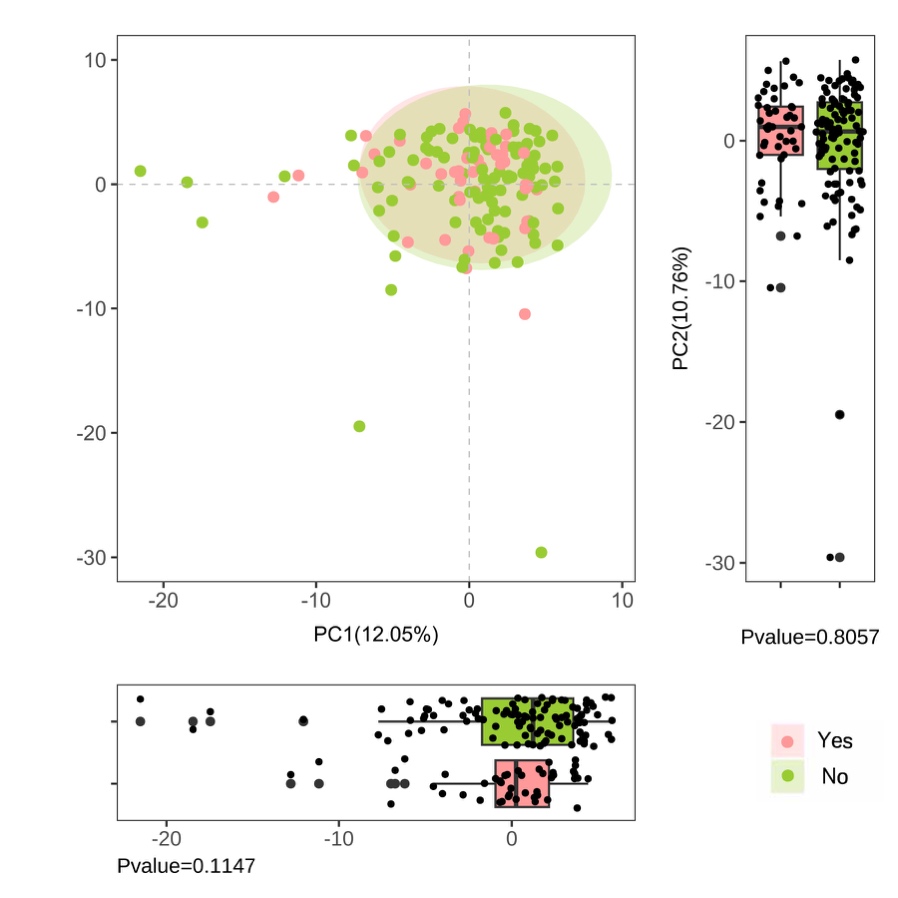


# Figure S1 Principal component analysis (PCA) for MSM grouped by whether performed rectal douching in the previous 3 months

A total of 149 participants were included in this analysis, with 45 reporting rectal douching in the previous 3 months and 104 not reporting it.

**
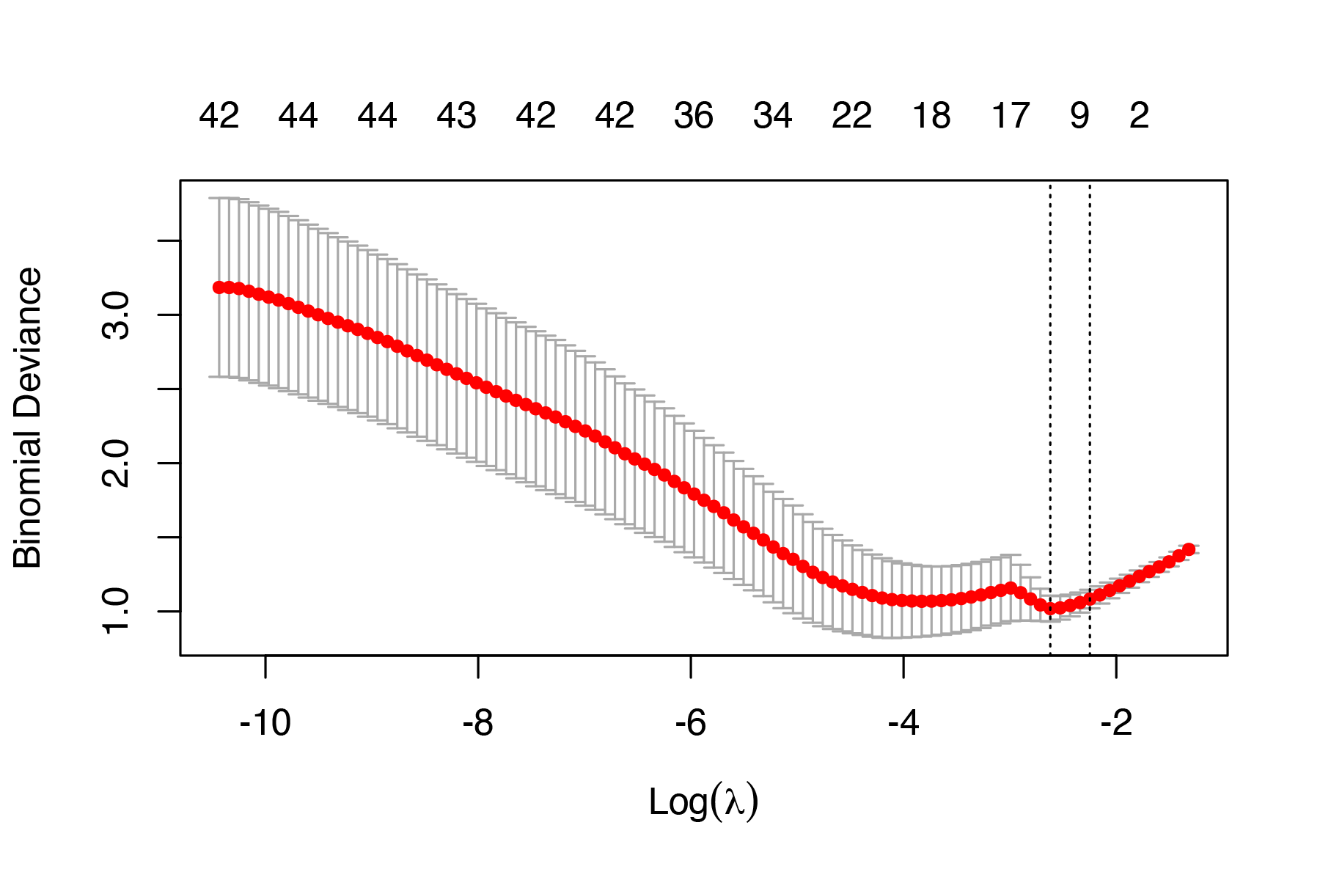
**

# Fig. S2. Identification of the optimal penalization coefficient (λ) (GIR vs HC)

Selection of the tuning parameter lambda (λ) using 10-fold cross validation. The dotted vertical lines were drawn at the optimal λ value based on the minimum criteria and 1 standard error of the minimum criteria. When λ = 0.07276316, a minimal root mean square error and 11 non-zero coefficients were obtained.


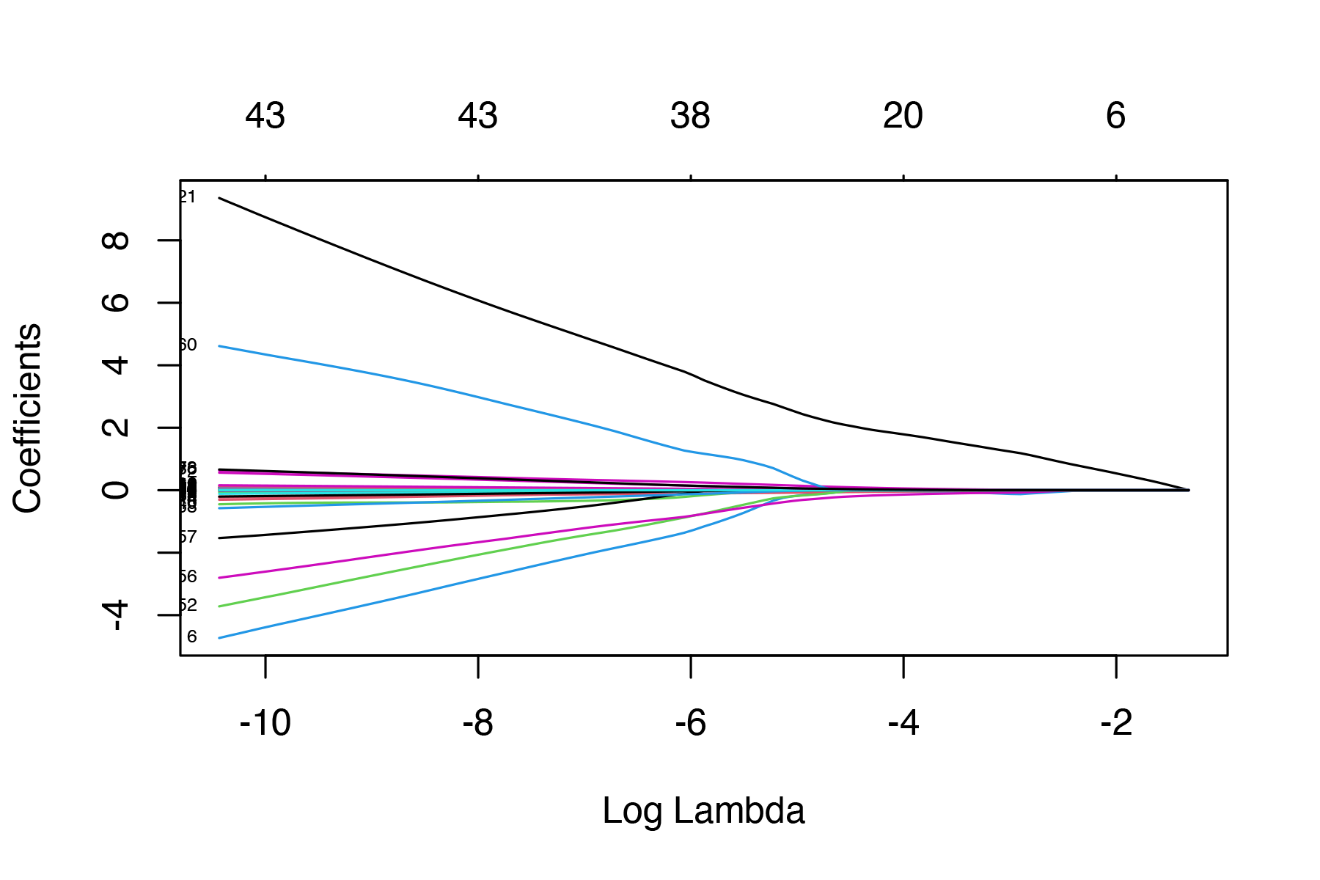


# Fig. S3. LASSO coefficient profiles (GIR vs HC)


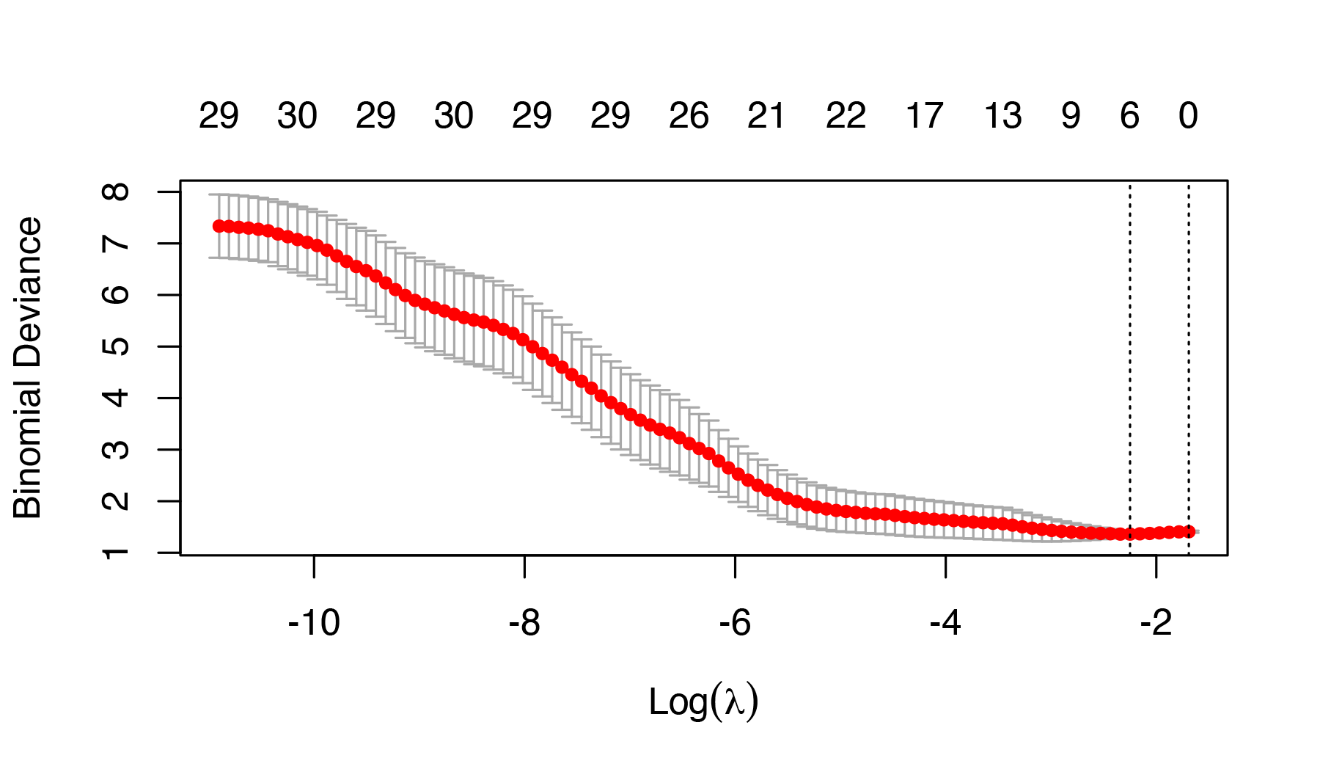


# Fig. S4. Identification of the optimal penalization coefficient (λ) (PIR vs HC)

Selection of the tuning parameter lambda (λ) using 10-fold cross validation. The dotted vertical lines were drawn at the optimal λ value based on the minimum criteria and 1 standard error of the minimum criteria. When λ = 0.1054818, a minimal root mean square error and 6 non-zero coefficients were obtained.


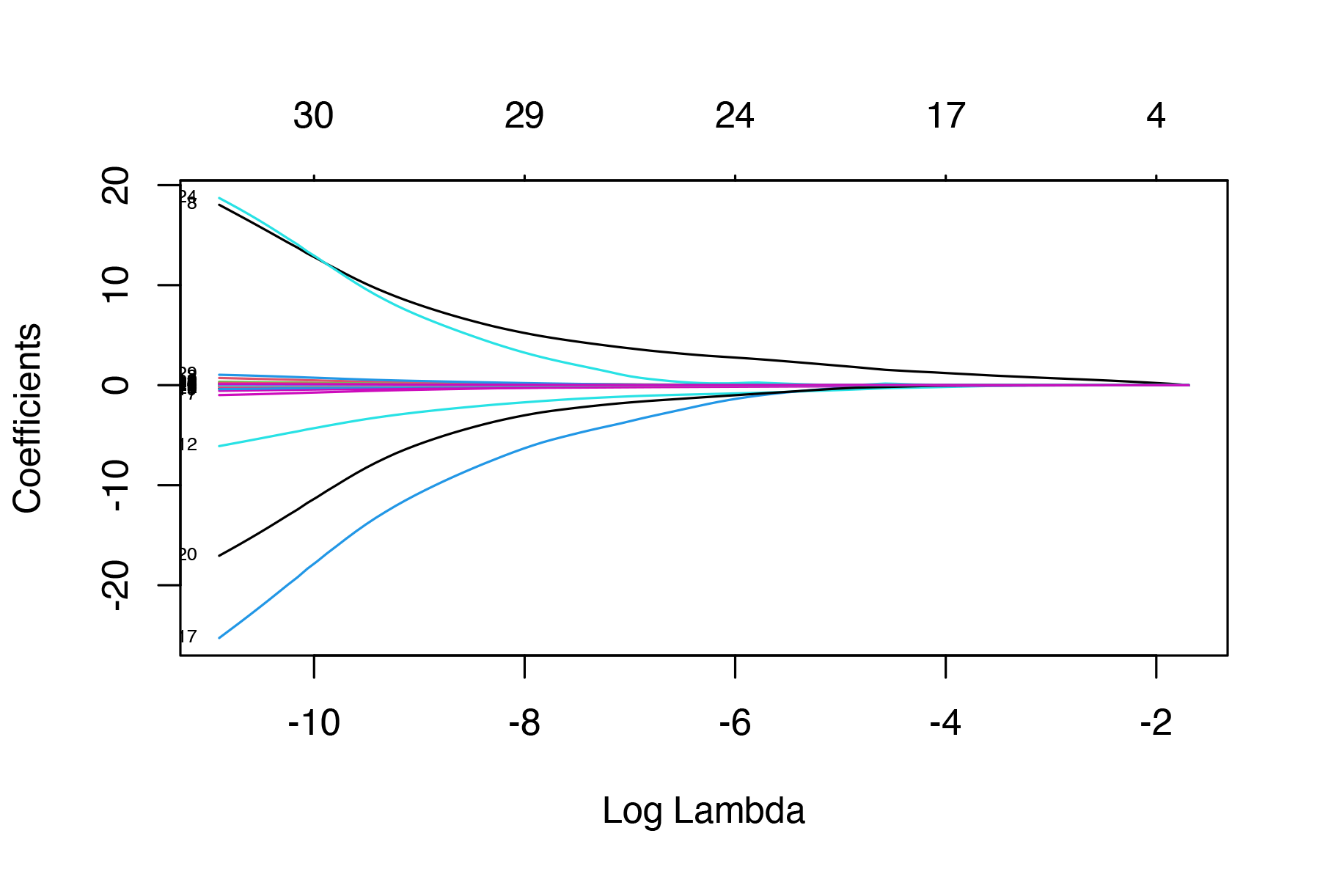


# Fig. S5. LASSO coefficient profiles (PIR vs HC)


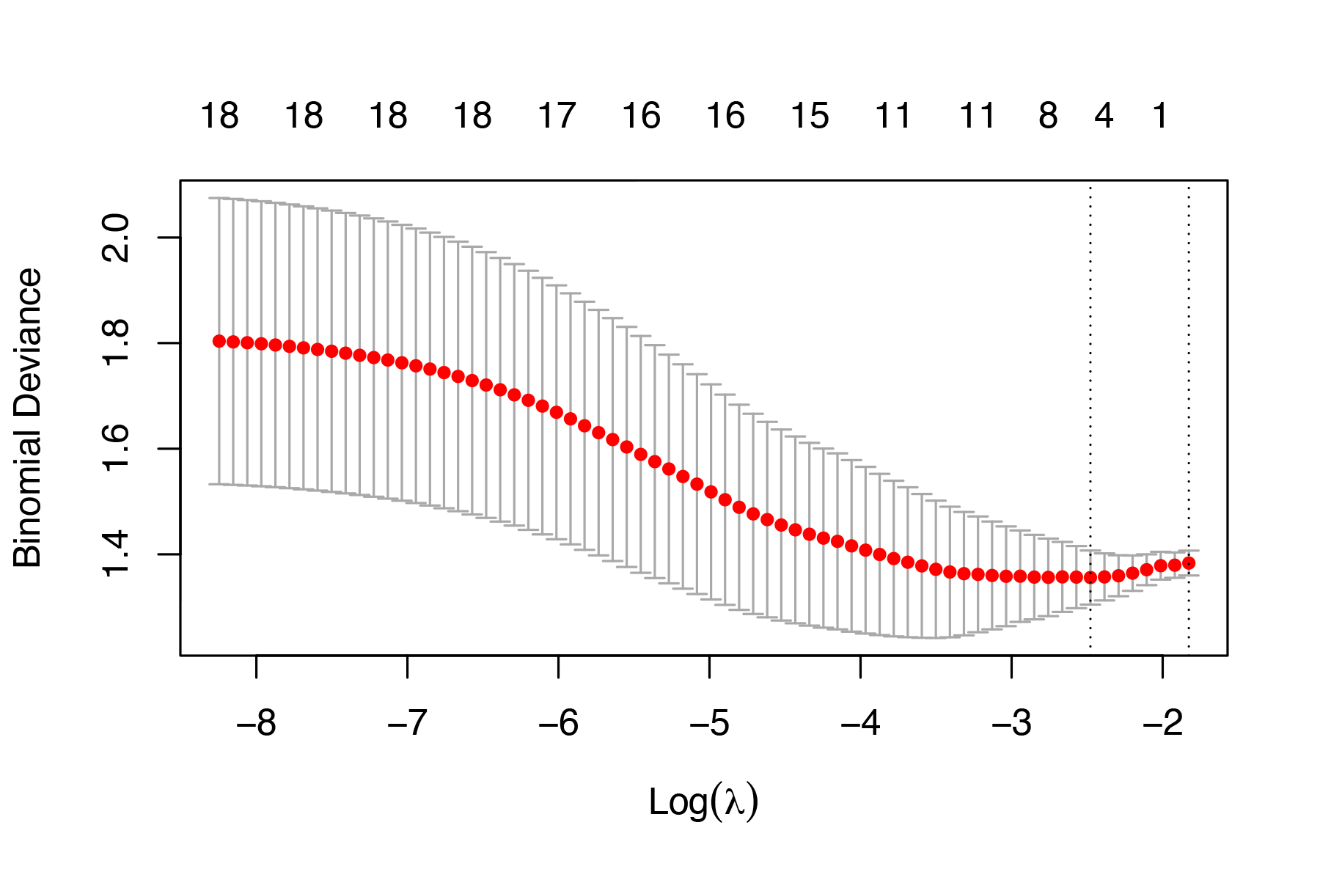


# Fig. S6. Identification of the optimal penalization coefficient (λ) (PIR vs GIR)

Selection of the tuning parameter lambda (λ) using 10-fold cross validation. The dotted vertical lines were drawn at the optimal λ value based on the minimum criteria and 1 standard error of the minimum criteria. When λ = 0.08390455, a minimal root mean square error and 4 non-zero coefficients were obtained


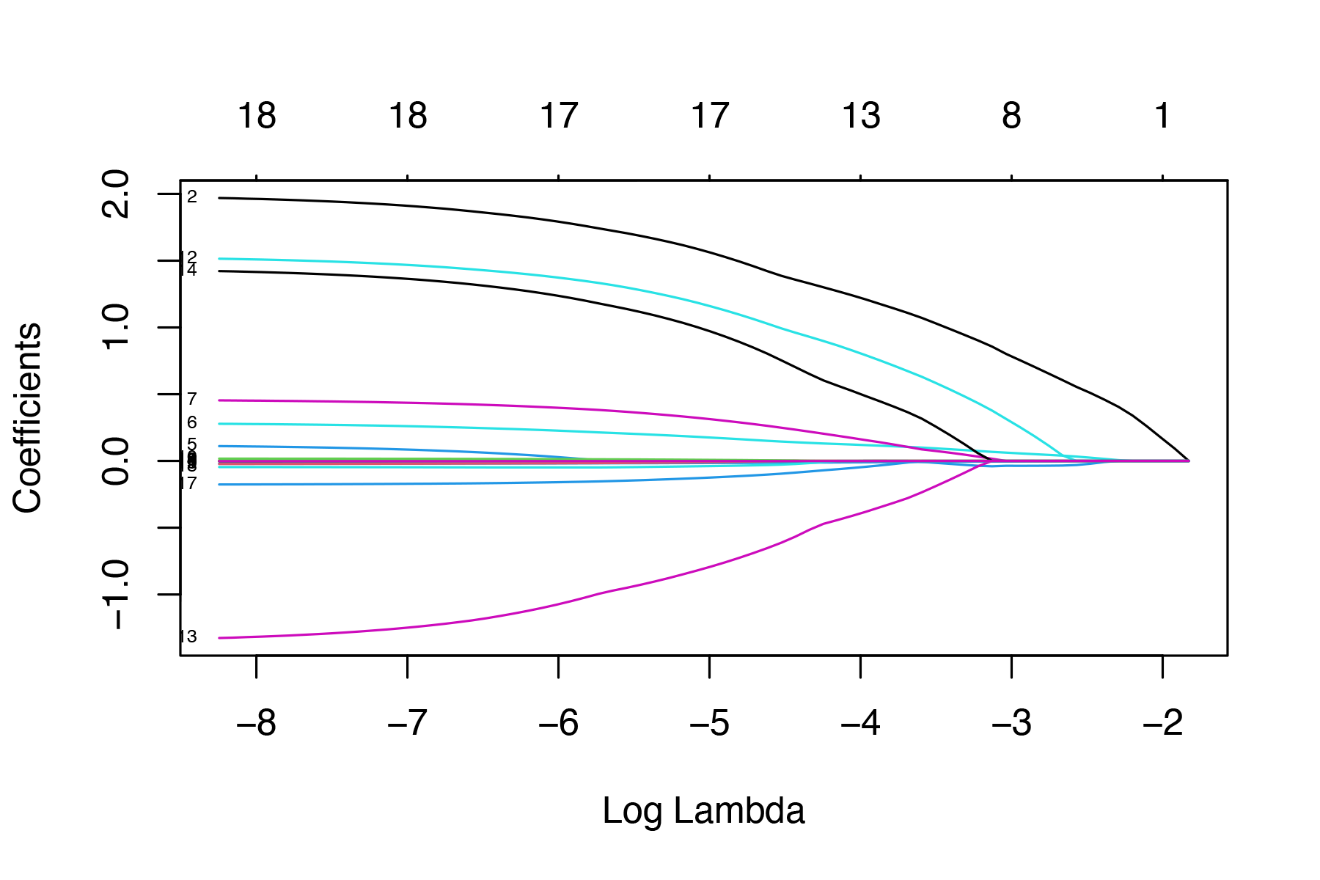


# Fig. S7. LASSO coefficient profiles (PIR vs GIR)
